# Genome-Wide Identification and Functional Analysis of the Calcineurin B-like Protein and Calcineurin B-like Protein-Interacting Protein Kinase Gene Families in Turnip (*Brassica rapa* var. *rapa*)

**DOI:** 10.3389/fpls.2017.01191

**Published:** 2017-07-07

**Authors:** Xin Yin, Qiuli Wang, Qian Chen, Nan Xiang, Yunqiang Yang, Yongping Yang

**Affiliations:** ^1^Key Laboratory for Plant Diversity and Biogeography of East Asia, Kunming Institute of Botany, Chinese Academy of ScienceKunming, China; ^2^Plant Germplasm and Genomics Center, Kunming Institute of Botany, Chinese Academy of SciencesKunming, China; ^3^Institute of Tibetan Plateau Research at Kunming, Kunming Institute of Botany, Chinese Academy of SciencesKunming, China; ^4^University of Chinese Academy of SciencesBeijing, China; ^5^School of Life Sciences, Yunnan UniversityKunming, China

**Keywords:** BrrCBL–BrrCIPK, expression profiles, preferential interactions, functional differentiation, turnip

## Abstract

The calcineurin B-like protein (CBL)–CBL-interacting protein kinase (CIPK) complex has been identified as a primary component in calcium sensors that perceives various stress signals. Turnip (*Brassica rapa* var. *rapa*) has been widely cultivated in the Qinghai–Tibet Plateau for a century as a food crop of worldwide economic significance. These CBL–CIPK complexes have been demonstrated to play crucial roles in plant response to various environmental stresses. However, no report is available on the genome-wide characterization of these two gene families in turnip. In the present study, 19 and 51 members of the BrrCBL and BrrCIPK genes, respectively, are first identified in turnip and phylogenetically grouped into three and two distinct clusters, respectively. The expansion of these two gene families is mainly attributable to segmental duplication. Moreover, the differences in expression patterns in quantitative real-time PCR, as well as interaction profiles in the yeast two-hybrid assay, suggest the functional divergence of paralog genes during long-term evolution in turnip. Overexpressing and complement lines in *Arabidopsis* reveal that *BrrCBL9.2* improves, but *BrrCBL9.1* does not affect, salt tolerance in *Arabidopsis*. Thus, the expansion of the BrrCBL and BrrCIPK gene families enables the functional differentiation and evolution of some new gene functions of paralog genes. These paralog genes then play prominent roles in turnip's adaptation to the adverse environment of the Qinghai–Tibet Plateau. Overall, the study results contribute to our understanding of the functions of the CBL–CIPK complex and provide basis for selecting appropriate genes for the in-depth functional studies of BrrCBL–BrrCIPK in turnip.

## Introduction

Plants are exposed to various adverse stress conditions during their growth and development process. To cope with numerous environmental stimuli, plants form a series of complex signal transduction mechanisms to perceive, transduce, and respond to different stresses. These responses can minimize the injury derived from adversity. Calcium is a ubiquitous intracellular secondary messenger in plants. This element regulates various plant growth and development processes, as well as abiotic and biotic stress responses (Xiong et al., 2002). In calcium regulatory networks, calcium-binding proteins that function as sensor molecules specifically receive cellular calcium signals and transmit these signals to downstream pathways (Roberts and Harmon, 1992; Li et al., 2016). The calcineurin B-like protein (CBL) belongs to a unique group of calcium sensors in plants. Hence, the calcium signature can bind to the elongation factor (EF) hand domains of the CBL proteins. Then, the CBL proteins bind to the NAF/FISL domain of the C-terminal of the CBL-interacting protein kinases (CIPKs). As such, the CBL proteins control the affinities and activities of numerous ion transporters (Kolukisaoglu et al., 2004; Luan, 2009). The CBL–CIPK network represents an example of a significantly divergent Ca^2+^-decoding paradigm unique to plants. The N-terminal MGCXXS/T motif of the CBL protein that mediates lipid modification by myristoylation and palmitoylation directs the CBL–CIPK system to an exact cellular target region. As a result, the CIPK is stimulated to phosphorylate the proper target proteins (Batistic et al., 2008; Batistic and Kudla, 2009). CBL1, -4, -5, -8, and -9 contained the shortest N-termini (Batistic et al., 2010; Sanchez-Barrena et al., 2013). Most of the genes contained the conserved MGCXXS/T motif that helped the CBLs to anchor in the membrane (Batistic et al., 2008; Weinl and Kudla, 2009). *Arabidopsis* CBL1, -4, -5, and -9 localize to the plasma membrane (Batistic et al., 2010).

The CBL and CIPK genes have been identified in many species, such as *Arabidopsis thaliana* (Kolukisaoglu et al., 2004), *Populus* (Zhang et al., 2008), *Oryza sativa* (Chen X. F. et al., 2011), *Zea mays* (Chen X. F. et al., 2011), *Brassica napus L*. (Zhang et al., 2014), *Triticum aestivum L*. (Sun et al., 2015), *Solanum melongena L*. (Li et al., 2016), *Physcomitrella patens* (Weinl and Kudla, 2009), and *Selaginella moellendorffii* (Weinl and Kudla, 2009). These studies have extended the analysis of CBL–CIPK interactions to the entire family of CBLs and CIPKs to discover functional pairs of CBLs and CIPKs. Previous studies reported that CBL–CIPK complexes are involved in mediating Ca^2+^ signals elicited by many kinds of stresses, such as low potassium content (*CBL1*/*CBL9*–*CIPK23* pathway) (Li et al., 2006; Xu et al., 2006; Cheong et al., 2007; Lee et al., 2007), high salt level (*CBL4*/*CBL10*–*CIPK24* pathway) (Qiu et al., 2002; Cheng et al., 2004), abscisic acid (ABA) exposure (*CBL9*–*CIPK3* pathway) (Pandey et al., 2004), and cold (*CBL1*–*CIPK7* pathway) (Huang et al., 2011). Studies on the CBL–CIPK network extensively demonstrated the interaction specificity and overlap among various members of the CBL and CIPK family. This finding reflects both the functional specificity and redundancy of the CBL and CIPK genes. For example, *Arabidopsis CBL1* and *CBL9* are similar in their amino-acid sequences and serve overlapping and specific functions (Albrecht et al., 2003; Pandey et al., 2004; Li et al., 2006; Xu et al., 2006; Cheong et al., 2007). This observation suggests that calcium sensors with high sequence similarities or a close evolutionary relationship may perform very distinct functions, and duplicated genes usually evolve some new functions. The overexpression and mutant analysis of CBLs and CIPKs have greatly enriched our understanding of the gene's functions (Thapa et al., 2011). These analyses have shown that the genes might develop tolerance to different stresses; thus, the plant harboring these genes can adapt to unfavorable conditions. For instance, *CBL1* expression is induced by wounding, high-salt conditions, cold, ABA exposure, and drought (Kudla et al., 1999; Cheong et al., 2003). The *cbl1* mutant is hypersensitive to drought, high-salt conditions, and hyperosmotic stress (Albrecht et al., 2003; Cheong et al., 2003). Meanwhile, *CBL9* is involved in ABA signaling and stress-induced ABA biosynthetic pathways (Pandey et al., 2004). *ThCBL9* overexpression conferred salt tolerance to transgenic *A. thaliana* (Sun et al., 2008). *cbl9* mutant plants are hypersensitive to ABA or high salt and mannitol (Pandey et al., 2004; Sun et al., 2008). Moreover, the double mutant *cbl1cbl9* has been generated to investigate the functions of the corresponding genes as that in the study of the *CBL1*/*9*–*CIPK23* complex involved in the regulation of potassium absorption. *cbl1cbl9* double mutants display defects in low-K conditions (Li et al., 2006; Xu et al., 2006; Lee et al., 2007), whereas *cbl1* and *cbl9* signaling mutants did not show phenotypic changes in K uptake (Cheong et al., 2003; Pandey et al., 2004).

Turnip (*Brassica rapa* var. *rapa*) belongs to a well-known plant family called Brassicaceae. The plant is a traditional crop highly adapted to the extreme environments of the Qinghai–Tibet Plateau and hold edible, feeding, and pharmaceutical values (Liang et al., 2006). Turnip undergoes a range of environmental stresses in its natural environment and has evolved a wide range of mechanisms to cope with these stresses. Accordingly, in this study, we explore the mechanisms and functions of the CBL–CIPK signaling system involved turnip's response to extreme environments. The functions of some members of CBL and CIPK have been studied in some species. However, the functions of many CBLs and CIPKs remain to be characterized to date. Moreover, the CBL–CIPK network in other plants remains fragmentary. To date, no systematic analysis of CBL or CIPK family genes has been reported in turnip. The manner by which specific CBL–CIPK complexes participate in turnip growth and development, as well as in response to abiotic and *jasmonic acid* (stress hormone) treatments, remains to be revealed. Thus, to analyze the mechanisms and functions of the CBL–CIPK signaling system in turnip in response to extreme environments is of great significance. In this present study, we carried out a genome-wide analysis and identified 19 BrrCBL and 51 BrrCIPK genes in turnip. We determined the genomic information; phylogenetic relationships; chromosome localization; and expression analysis for different tissues (root, stem, leaf, and flower), different development stages (early stage [ES], cortex-splitting stage [CSS], and root-thickening stage [RTS]), and stress treatments (drought, cold, NaCl, and methyl jasmonate [MeJA]) related to the two families of genes and proteins. We also investigated the BrrCBL–BrrCIPK interactions, determined their effects on salt tolerance, and performed overexpression and complementation analyses of *BrrCBL9.1* and -*9.2*. Functional divergence was determined between paralog genes in the two families of genes and proteins. The results provide a solid foundation and may help in further elucidating how the turnip CBL–CIPK network enables the integration of multiple signals from the plant's environment and coordinate downstream responses to stresses in the Qinghai–Tibet Plateau. The findings also offer a useful framework for additional functional studies in this area.

## Materials and methods

### Identification and structure analyses of the CBL and CIPK gene family in turnip

The 10 *Arabidopsis* CBL and 26 CIPK genes were downloaded from TAIR (http://www.arabidopsis.org) as queries to search against turnip genomes (http://www.bioinformatics.nl/brassica/index.html?data=bras_tp%2Fdata&loc=A03%3A22821424..22821483&tracks=DNA%2CTranscripts%2CGenes&highlight=). The domains and functional sites in each protein were examined with ps_scan.pl. All CBL protein sequences containing the EF-hand calcium-binding domains (PS50222), as well as all CIPK protein sequences with protein kinase domains (PS50011) and the NAF/FISL motif (PS50816) were extracted as candidates. All candidates were used to search against the GenBank nonredundant protein database. Homology analysis between turnip and *Arabidopsis* was conducted using DNAMAN software. Physicochemical parameters including the MW, theoretical PI, grand average of hydropathicity, and the number of amino acids were calculated using the ProtParam tool of ExPaSy (http://web.expasy.org/protparam/; Gasteiger et al., 2005). Putative EF-hand was predicated using a simple modular architecture research tool (http://smart.embl-heidelberg.de/). Myristoylaton and palmitoylation motifs were predicted using PlantsP (http://mendel.imp.ac.at/myristate/SUPLpredictor.htm) and CSS-Palm 3.0 software (Ren et al., 2008), respectively. The diagram of the intron/exon structures of BrrCBL and BrrCIPK was analyzed using the online Gene Structure Display Server (http://gsds.cbi.pku.edu.cn/; Hu et al., 2015). Subsequently, the MEME program was used to search for conserved motifs in the turnip BrrCBL and BrrCIPK protein sequences (Bailey et al., 2006).

### Phylogenetic analysis and chromosomal location, divergence time estimation

The BrrCBL and BrrCIPK protein sequences were aligned using the MAFFT version 7 program, and phylogenetic trees were constructed using the MEGA 5.0 software with the neighbor-joining method and the 1,000 bootstrap test replicates (Tamura et al., 2011).

To map the locations of BrrCBL and BrrCIPK genes in turnip, the chromosomal distribution of turnip genomic sequences were generated by circus software (http://www.circos.ca/software/download/circos/). Then, two types of gene duplications were recognized, namely, segment duplication and tandem duplication (Flagel and Wendel, 2009). The synonymous (Ks) and nonsynonymous (Ka) substitution rates were estimated by the codeml program of PAML4 (Yang, 2007). The divergence time (T) of BrrCBL and BrrCIPK gene pairs was calculated as T = Ks/2λ (divergence rates λ = 1.5 × 10^−8^ for *Arabidopsis*; Cao et al., 2011).

### Subcellular localization and confocal laser scanning microscopy

The coding region (CDS) of BrrCBL genes was cloned and fused to a binary vector pRI101-AN DNA, with a *green fluorescent protein* (GFP) and driven by the Cauliflower mosaic virus (CaMV) 35S promoter, forming a *35S:BrrCBL*-GFP construct. After confirmation by sequencing, these constructs were separately transferred into *Agrobacterium tumefaciens* EHA105 by electroporation for infiltration into leaves of *Nicotiana benthamiana* (Wydro et al., 2006). For the control, we tested the subcellular localization of GFP alone in the leaf cells of *N. benthamiana*. Freshly made transformed Agrobacteria cell cultures were resuspended in media containing 10 mM MES–KOH (pH 5.6), 10 mM MgCl_2_·6H_2_O, and 100 μM/L acetosyringone (AS, Sigma), adjusted to an OD_600_ of 0.6–0.8. Next, 3 mL of culture was obtained by sterile single-use syringes to inject into the abaxial air space of *N. benthamiana* leaves. The leaf section near the injection site was squashed 3 days after infiltration. Fluorescence images were detected using a laser-scanning confocal microscope (Olympus Optical Co. Ltd., Tokyo, Japan). GFP was excited with a 488 nm laser line, and emissions were captured using a 505–530 band-pass filter.

### Different expression profile of BrrCBL and BrrCIPK genes

The estimated expression profiles expressed in fragments per kilobase of exon model per million mapped reads values for each BrrCBLs and BrrCIPKs from different developmental stages were obtained from RNA-Seq data in the reanalyzed turnip (Li et al., 2015). Samples were obtained in the early stage before cortex splitting (ES1 and ES2), cortex-splitting stage (CSS1 and CSS2), and secondary root-thickening stage (RTS1 and RTS2) in turnip. These stages were described in detail in a previous study (Li et al., 2015). The normalization and hierarchical clustering analysis of gene expression patterns were performed on the basis of Pearson coefficients with average linkage using the Genesis software (version 1.7.1; Sturn et al., 2002).

### Plant material, growth condition, and stress treatments

Seeds of turnip were grown in soil pots and 1/4 Hoagland's nutrient solution (pH 5.5) under controlled conditions (28°C day/25°C night cycle, relative humidity of 75–80%, 200 mmol photons m^−2^ s^−1^ light intensity). After 3 weeks of germination, drought stress was executed by withholding water for 7 days. For MeJA, high salinity, and cold stress, seeding was exposed to MeJA (1 mM), NaCl (100 mM), and cold temperature (4°C). The roots and leaves of seedlings were harvested after treatment for 3 h, with 0 h as the control. The samples, including the root, stem, leaf, and flower, were harvested at the flowering stage. All samples were immediately frozen in liquid nitrogen and then stored at −80°C for RNA extraction.

### RNA isolation and quantitative real-time PCR (qRT-PCR) analysis

Total RNA samples were isolated using the Eastep® Super Total RNA Extraction Kit (Promega, Madison, WI, USA). RNA was quantified by NanoDrop1000 (NanoDrop Technologies, Inc.) with integrity checked on 0.8% agarose gel. Approximately 5 μg RNA was reverse transcribed using the GoScript Reverse Transcription System (Promega, Madison, WI) to generate cDNA. qRT-PCR was conducted in triplicate with different cDNAs synthesized from three biological replicates of different tissues and treatments using FastStart Universal SYBR Green Master (Rox, Roche, Indianapolis, IN, USA) and a 7,500 Sequence Detection System (Applied Biosystems, USA). The reaction parameters for thermal cycling were as follows: 95°C for 10 min, followed by 40 cycles of 94°C for 5 s, and 60°C for 15 s. *B. rapa* tubulin beta-2 chain-like (LOC103873913) was amplified as an internal control. The primers used for qRT-PCR are listed in Supplementary Table 1. The relative gene expression levels were obtained by dividing the extrapolated transcript levels of the target genes by the levels of the internal control from the same sample. The results were obtained from the comparison of the treatment with the control using independent-samples *T*-test. Statistical analysis was performed using the software IBM SPSS Statistics 20.0.

### Yeast two-hybrid (Y2H) and bimolecular fluorescence complementation (BiFC) assays

Y2H assays were conducted using the MatchMaker Y2H system (http://www.clontech.com/). The CDS regions of BrrCBL and BrrCIPK genes were first subcloned into pGADT7 and pGBKT7 vectors, respectively. The primers used for vector construction are listed in Supplementary Table 1. Then, the plasmids of BrrCBL and BrrCIPK were sequentially transformed into yeast strain AH109 through the lithium acetate method following the protocol described in the Yeast Protocols Handbook (Clontech). The transformed yeast cells were grown on medium as follows: (1) lacking leucine and tryptophan SD−Trp−Leu to serve as a positive control for transformation and loading; (2) lacking leucine, tryptophan, and histidine SD−Trp−Leu−His to test for PPIs under low stringency, and (3) lacking leucine, tryptophan, histidine, and adenine SD−Trp−Leu−His−Ade to test for interactions under stringent conditions. Cell growth was recorded at 48 h intervals over the course of 6 days. The method of assessment of the interaction strength between sets of proteins was as follows (Kleist et al., 2014). Red boxes indicate vigorous growth on -LTHA plates. Orange boxes indicate weak growth on -LTHA plates. Yellow boxes indicate robust growth on -LTH plates but no growth on -LTHA plates. Pale yellow boxes indicate weak growth on -LTH plates but with each CBL–CIPK interaction conferring superior growth to that in the empty vector control. Gray boxes indicate only growth on -LT plates.

For BiFC assays, the CDS regions of selected BrrCBL and BrrCIPK genes were sub-cloned into 35s-SPYCE and 35s-SPYNE vectors, respectively. The primers used for vector construction are listed in Supplementary Table 1. The fusion construct was transferred into *A. tumefaciens* strain EHA105, then into tobacco leaves by *Agrobacterium* infiltration (Wydro et al., 2006). Infiltrated leaves were observed with a laser scanning confocal microscope (Olympus Optical Co. Ltd., Tokyo, Japan).

### *Arabidopsis* transformation and treatments

The full-length coding sequences of *BrrCBL9.1* and *BrrCBL9.2* were each inserted into a binary vector pRI101-AN DNA fused with a GFP and driven by Cauliflower mosaic virus (CaMV) 35S promoter. Consequently, a *35S: BrrCBL*-GFP construct was obtained. *Arabidopsis* plants were transformed using the floral-dip method with the *A. tumefaciens* strain EHA105 (Clough and Bent, 1998). The assayed plants (overexpression, *cbl9* mutant, complemented lines, and WT) were grown on 1/2 solid Murashige and Skoog (MS) medium containing NaCl (100 mM) for stress analysis. After 8 days, the seedling root lengths were measured. For expression analysis of corresponding BrrCIPK genes specially interacted with BrrCBL9.1 and BrrCBL9.2 in overexpression, *cbl9* mutant, and complemented lines, the assayed plants were grown on MS medium for about 10 days. Then they were transferred to soil in a growth chamber (photoperiod of 16-h light/8 h darkness) at 21°C. After 2 weeks of germination, seedings were exposed to NaCl (100 mM) and were harvested after treatment for 3 h, with 0 h as the control. All samples were immediately frozen in liquid nitrogen and then stored at −80°C for RNA extraction.

## Results

### Identification and characterization of BrrCBL and BrrCIPK genes

We conducted a genome-wide identification of CBLs and CIPKs in turnip. Accordingly, we used *Arabidopsis* 10 CBL and 26 CIPK CDS sequences (Zhang et al., 2014) as queries to search against the published genome of turnip. After the search, we identified 19 CBLs and 51 CIPKs, the names assigned to these genes, and their phylogenies and sequence similarities to corresponding individual AtCBLs and AtCIPKs (Tables 1, 2). The full-length turnip CBL proteins were 194 (BrrCBL5) to 249 (BrrCBL10.1) amino acids long, except BrrCBL3.3 with 449 amino acids (Table 1). All the BrrCBLs contained four EF-hand motifs, which provide the structural basis for calcium binding. BrrCBL1.1, -1.2, -4.1, -4.2, -4.3, -5, -9.1, and -9.2 proteins each harbored a conserved N-myristoylation and palmitoylation motif (MGCXXS/T) that may be responsible for membrane association (Batistic et al., 2008). The BrrCIPKs identified in our study ranged in isoelectric points (PIs) from 5.49 (BrrCIPK16) to 9.28 (BrrCIPK23.1), with coding sequences of 352–524 amino acids (Table 2). These ranges suggest the diversity of the biochemical properties of BrrCIPKs. Among the identified BrrCBL and BrrCIPK genes, the CDS of 15 BrrCBLs and 47 BrrCIPKs were successfully amplified by PCR from cDNA prepared from turnip as the template using available primers (Supplementary Table 1). We further predicted the localization of 15 BrrCBL proteins by confocal laser microscopy (Supplementary Table 1). The BrrCBL1.1, -1.2, -2.2, -3.2, -4.3, -5, -8, -9.1, and -9.2–GFR fusion proteins each emitted a green fluorescent signal in the plasma membranes and nuclei of the epidermal cells of *N. benthamiana* leaves. By contrast, BrrCBL2.1, -3.1, -4.2, -6, -10.2, and -10.4–GFR were detected in plasma membranes. Although, lacking myristoylation and palmitoylation motifs, BrrCBL3.2 and BrrCBL8 localized in the membrane. The dynamic localization of CIPKs was determined by their specific CBL partners (Batistic et al., 2010; Batistic and Kudla, 2012). We did not analyze the subcellular localization of the CIPK protein.

**Table 1:** Turnip CBL genes identified and their characteristics.

**Gene name**	***Arabidopsis* ortholog**	**Identity/%**	**Gene locus**	**MW(Da)**	**PI**	**GRAVY**	**No. of amino acids**	**No. of EF-hand**	**Myristoylaton motif**	**Palmitoylation prediction**
BrrCBL1.1	AtCBL1	97.65	A01:4433745..4435654 (+ strand)	24489.8	4.63	−0.172	213	4	Y	Y
BrrCBL1.2		95.31	A03:22819854..22821724 (+ strand)	24603.0	4.64	−0.173	213	4	Y	Y
BrrCBL2.1	AtCBL2	97.35	A02:7317842..7319176 (− strand)	25817.4	4.89	−0.217	226	4	N	Y
BrrCBL2.2		84.87	A03:5542770..5543954 (− strand)	25135.7	4.96	−0.227	220	4	N	Y
BrrCBL3.1	AtCBL3	93.48	A01:9368109..9369455 (− strand)	25783.2	4.78	−0.227	226	4	N	Y
BrrCBL3.2		56.67	A01:9370155..9371547 (− strand)	27891.5	5.02	−0.432	248	4	N	N
BrrCBL3.3^a^		45.03	A03:26225550..26228758 (− strand)	50486.0	5.72	−0.186	449	4	N	Y
BrrCBL4.1^a^	AtCBL4	89.19	A06:16916876..16918301 (− strand)	25582.3	5.02	−0.282	221	4	Y	Y
BrrCBL4.2		84.23	A02:25285057..25286247 (+ strand)	25311.0	4.78	−0.214	221	4	Y	Y
BrrCBL4.3		79.28	A01:9131128..9132335 (− strand)	22520.5	5.13	−0.238	195	4	Y	Y
BrrCBL5	AtCBL5	78.05	A10:10371156..10373185 (− strand)	22430.5	5.23	−0.332	194	4	Y	Y
BrrCBL6	AtCBL6	76.42	A01:11115972..11117248 (+ strand)	26206.2	5.99	−0.21	227	4	N	Y
BrrCBL8	AtCBL8	91.59	A09:6789352..6790915 (+ strand)	24630.2	5.32	−0.243	214	4	N	N
BrrCBL9.1	AtCBL9	97.65	A02:19306250..19307973 (− strand)	24350.6	4.62	−0.17	213	4	Y	Y
BrrCBL9.2		95.31	A09:15793572..15795358 (+ strand)	24296.5	4.62	−0.236	213	4	Y	Y
BrrCBL10.1^a^	AtCBL10	82.49	A01:2309900..2311454 (− strand)	28735.0	4.89	−0.019	249	4	N	Y
BrrCBL10.2		78.21	A01:2310578..2312098 (− strand)	27455.6	5.05	−0.008	238	4	N	Y
BrrCBL10.3^a^		70.31	A01:2309444..2310772 (− strand)	24335.9	4.67	−0.075	211	4	N	Y
BrrCBL10.4		65.76	A03:28734206..28735230 (+ strand)	23718.1	4.37	−0.067	207	4	N	Y

a *Those genes that were not amplified in this study; MW, molecular weight; PI, isoelectric point; GRAVY, Grand average of hydropathicity; Myristoylaton motif, predicted by PlantsP, http://mendel.imp.ac.at/myristate/SUPLpredictor.htm; Palmitoylation prediction, Predicted by CSS-Palm 3.0 software, threshold: high, http://csspalm.biocuckoo.org/*.

**Table 2:** Turnip CIPK genes identified and their characteristics.

**Gene name**	***Arabidopsis* ortholog**	**Identity/%**	**Gene locus**	**MW(Da)**	**PI**	**GRAVY**	**No. of amino acids**
BrrCIPK1.1	AtCIPK1	91.70	A05:18922511..18925933 (+ strand)	50060.26	6.08	−0.373	446
BrrCIPK1.2		91.69	A03:17689534..17693160 (− strand)	49877.06	6.17	−0.379	443
BrrCIPK2.1	AtCIPK2	80.47	A10:15971237..15972634 (+ strand)	52675.29	7.59	−0.436	466
BrrCIPK2.2^a^		72.15	A02:2425631..2426950 (− strand)	49660.32	6.78	−0.394	439
BrrCIPK2.3		72.15	A10:15973585..15974907 (+ strand)	49378.29	9.03	−0.382	440
BrrCIPK3	AtCIPK3	94.46	A04:11827818..11830154 (− strand)	50253.49	6.82	−0.534	440
BrrCIPK4.1	AtCIPK4	78.03	A01:12509499..12516168 (+ strand)	48070.31	8.43	−0.272	432
BrrCIPK4.2^a^		70.47	A01:12530319..12531650 (+ strand)	49859.51	8.41	−0.295	443
BrrCIPK5	AtCIPK5	81.6	A10:14753941..14755201 (+ strand)	49526.82	6.14	−0.329	434
BrrCIPK6.1	AtCIPK6	95.25	A01:3141480..3142964 (− strand)	49113.48	8.81	−0.283	441
BrrCIPK6.2		95.02	A08:13459208..13469285 (− strand)	49175.55	8.69	−0.284	441
BrrCIPK6.3		90.48	A08:13458005..13459303 (− strand)	48522.03	9.01	−0.298	432
BrrCIPK6.4		87.7	A03:27998691..28000004(+ strand)	49020.53	8.98	−0.302	437
BrrCIPK7.1	AtCIPK7	85.08	A01:19820449..19821693 (− strand)	46273.4	9.21	−0.227	414
BrrCIPK7.2		83.72	A05:14807450..14808704 (− strand)	47448.81	8.48	−0.206	424
BrrCIPK8	AtCIPK8	94.84	A01:7773361..7776370 (+ strand)	50402.13	8.49	−0.207	446
BrrCIPK9.1	AtCIPK9	92.39	A09:36803277..36806394 (− strand)	49822.14	8.33	−0.393	445
BrrCIPK9.2		89.93	A10:4494425..4508468 (− strand)	48122.21	6.5	−0.365	427
BrrCIPK10.1	AtCIPK10	85.63	A10:8337171..8335783 (− strand)	52778.51	8.29	−0.54	462
BrrCIPK10.2		78.33	A02:6403330..6404661 (+ strand)	50903.74	8.9	−0.465	443
BrrCIPK11.1	AtCIPK11	84.75	A05:7363988..7365314 (+ strand)	49849.38	8.47	−0.325	443
BrrCIPK11.2		82.23	A04:13512421..13517107 (− strand)	49403.9	8.62	−0.283	437
BrrCIPK11.3		81.24	A03:7056590..7057869 (− strand)	48005.45	7.99	−0.234	427
BrrCIPK11.4		69.98	A05:20183381..20184484 (− strand)	41497.64	6.49	−0.322	367
BrrCIPK12.1	AtCIPK12	90.91	A01:5077746..5079156 (− strand)	55251.38	6.75	−0.266	494
BrrCIPK12.2		90.02	A08:10633529..10634939 (− strand)	55002.29	6.75	−0.249	488
BrrCIPK13.1	AtCIPK13	84.6	A04:15057804..15059342 (− strand)	57897.04	7.91	−0.268	512
BrrCIPK13.2		83.56	A05:5492184..5493662 (+ strand)	55244.17	8.99	−0.207	492
BrrCIPK13.3		79.64	A05:5485948..5487402 (+ strand)	54729.53	9.1	−0.213	486
BrrCIPK14	AtCIPK14	67.18	A02:1441346..1442455 (+ strand)	41915.23	7.19	−0.228	369
BrrCIPK15	AtCIPK15	63.85	A02:1446210..1447214 (− strand)	38235.65	5.99	−0.485	338
BrrCIPK16	AtCIPK16	85.99	A04:11266272..11268653 (− strand)	52348.96	5.49	−0.393	461
BrrCIPK17.1	AtCIPK17	81.71	A06:2357382..2363949 (+ strand)	47310.47	7.6	−0.194	425
BrrCIPK17.2		79.86	A08:3290707..3294907 (+ strand)	46919.14	7.59	−0.261	418
BrrCIPK17.3		73.21	A05:12344276..12348644 (+ strand)	44907.99	8.01	−0.192	399
BrrCIPK17.4^a^		66.44	A06:2366510..23691189 (+ strand)	47354.61	8.02	−0.154	425
BrrCIPK18	AtCIPK18	87.17	A08:16669033..16670623 (+ strand)	58887.96	7.54	−0.298	524
BrrCIPK19	AtCIPK19	89.05	A06:24828419..24829861 (− strand)	54290.75	8.93	−0.249	480
BrrCIPK20.1	AtCIPK20	89.07	A02:18174974..18176281 (− strand)	49858.82	9.22	−0.457	435
BrrCIPK20.2^a^		66.59	A06:24826182..24827465 (+ strand)	40057.09	8.87	−0.437	352
BrrCIPK21.1	AtCIPK21	93.03	A10:8022273..8024192 (− strand)	46241.58	8.68	−0.137	415
BrrCIPK21.2		92.79	A03:5119999..5122061 (+ strand)	46141.54	8.75	−0.121	415
BrrCIPK22.1	AtCIPK22	82.64	A03:9106897..9108189 (− strand)	48732.32	9.11	−0.366	430
BrrCIPK22.2		81.32	A05:3392707..3393990 (+ strand)	48342.69	9.15	−0.354	427
BrrCIPK23.1	AtCIPK23	91.96	A07:5680363..5683070 (− strand)	53391.34	9.28	−0.389	482
BrrCIPK23.2		90.04	A09:220102819..22013068 (− strand)	51920.76	9.17	−0.333	467
BrrCIPK23.3		71.58	A08:16341583..16336015 (− strand)	41900.98	8.08	−0.302	376
BrrCIPK24	AtCIPK24	87.64	A04:7485135..7487998 (− strand)	51582.54	9.13	−0.278	453
BrrCIPK25	AtCIPK25	75.2	A06:17403211..17404436 (− strand)	51679.66	8.68	−0.305	456
BrrCIPK26.1	AtCIPK26	91.16	A10:9976095..9979201 (− strand)	49931	7.63	−0.502	441
BrrCIPK26.2		85.03	A02:5263451..5266730 (+ strand)	49806.75	7.63	−0.511	441

a *Those genes that were not amplified in this study; MW, molecular weight; PI, isoelectric point; GRAVY, Grand average of hydropathicity*.

### Phylogenetic relationships and gene structural analyses of the BrrCBLs and BrrCIPKs

**Figure 1:**
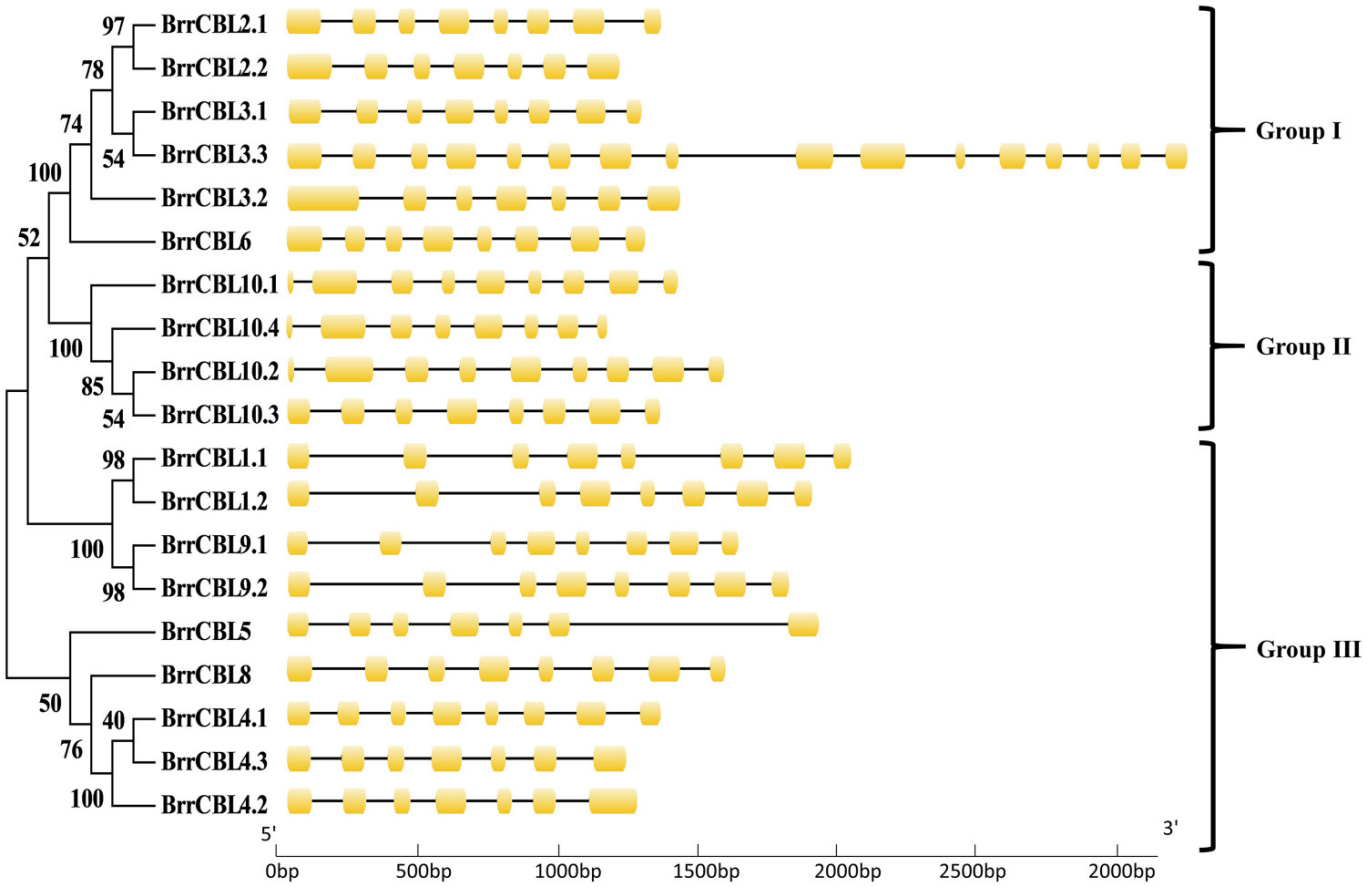
Phylogenetic relationship of Turnip CBL proteins. The BrrCBL protein sequences were aligned using the MAFFT version 7 program, and phylogenetic trees were constructed using the MEGA 5.0 software with the neighbor-joining method and the 1,000 bootstrap test replicates. The tree can be divided into three major clades (Group I – Group III).

To gain insights into the phylogenetic analysis of both CBL and CIPK families, we generated two phylogenetic trees for each family. The BrrCBL proteins were divided into three groups, namely, groups I, II, and III (Figure 1). We also compared the evolutionary relationship between turnip and *Arabidopsis* in terms of the CBLs (Supplementary Figure 2A). All BrrCBLs were clustered closely with AtCBL orthologs. However, *CBL7* was present in *Arabidopsis* but absent in turnip. To analyze the structural characteristics of the BrrCBLs, we mapped gene structures, including exons and introns, on the basis of the turnip genome sequence. The results showed that all the BrrCBLs were intron rich, with six to eight introns (except *BrrCBL3.3* with the 15 introns). Hence, the exon/intron numbers of the CBL gene potentially vary. Moreover, we analyzed the EF-hand motifs for the BrrCBL proteins (Supplementary Figure 3). BrrCBL1, -3, -4, -5, and -8 possessed no canonical EF hand, whereas BrrCBL6, -7, and -10 possessed one and BrrCBL1 and -9 harbored two canonical EF hands. The space of each EF-hand motif was absolutely conserved in the BrrCBL proteins. Up to 23 amino acids were observed to lie between EF1 and EF2, 25 amino acids between EF2 and EF3, and 32 amino acids between EF3 and EF4.

**Figure 2:**
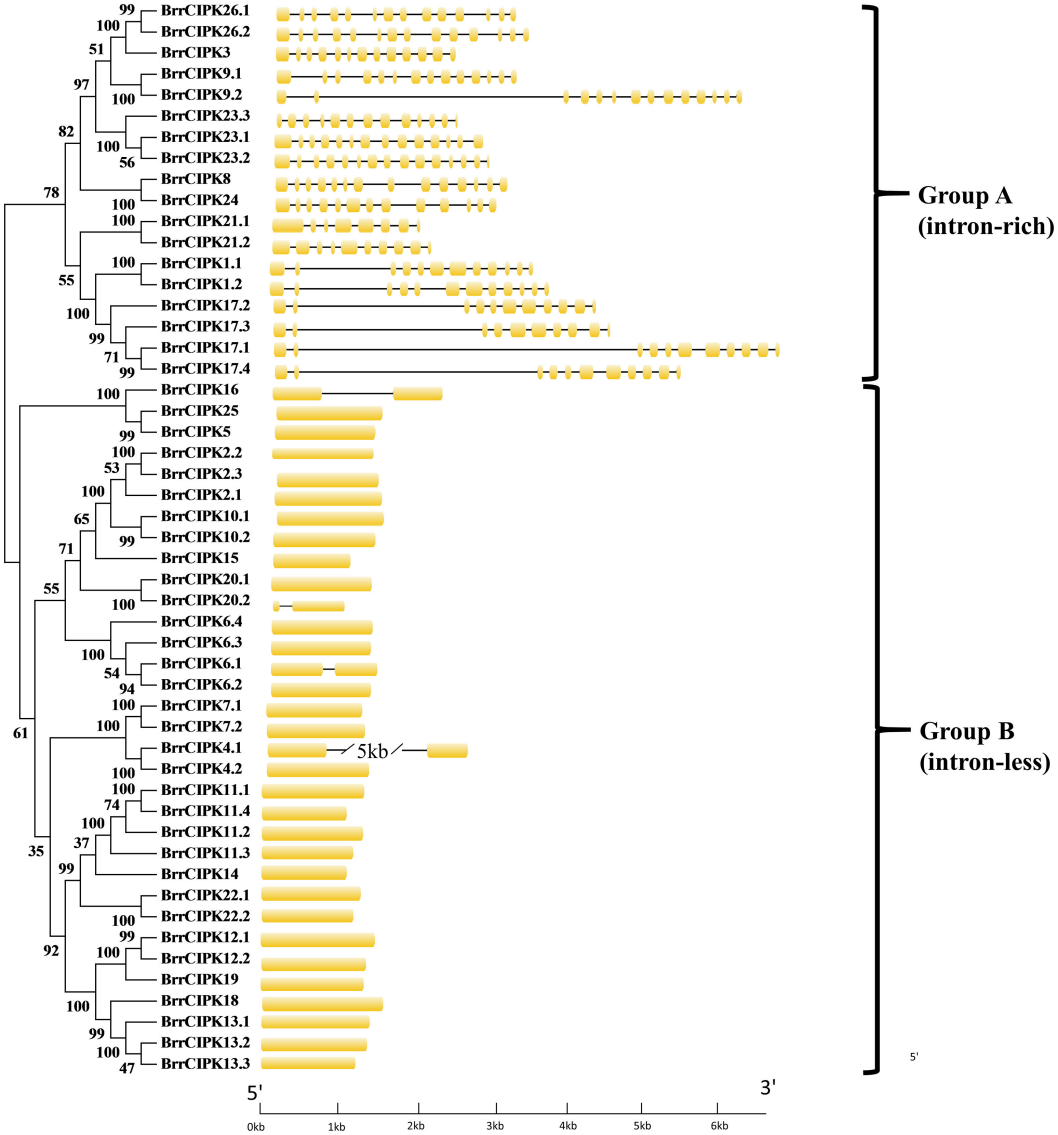
Phylogenetic relationship of Turnip CIPK proteins. The BrrCIPK protein sequences were aligned using the MAFFT version 7 program, and phylogenetic trees were constructed using the MEGA 5.0 software with the neighbor-joining method and the 1,000 bootstrap test replicates. The tree can be divided into two major clades (Group A and Group B).

As expected, the phylogenetic tree divided the BrrCIPKs into two groups, namely, group A (intron-rich) (18 BrrCIPKs) and group B (intron-less) (33 BrrCIPKs); this finding was consistent with previous evolutionary analyses (Kolukisaoglu et al., 2004) (Figure 2). The structural differences in the BrrCIPKs may allow BrrCIPK genes to function differently because the functional structural gene domains determine the gene's function (Albrecht et al., 2001). All of the 51 BrrCIPKs were clustered closely with AtCIPK orthologs (Supplementary Figure 2B). In the *Arabidopsis* CIPKs (Batistic and Kudla, 2004), all BrrCIPKs contained domain structures similar to that of AtCIPK24, including an N-terminal protein kinase catalytic (PKC) domain and a C-terminal regulatory domain (NAF/FISL motif) was identified (Supplementary Figure 4A). Previous studies showed that the PKC domain in the N-terminal and a short motif called NAF/FISL motif located in the C-terminal regulatory domain were necessary and sufficient for mediating interactions with CBLs (Albrecht et al., 2001; Guo et al., 2001; Hashimoto et al., 2012). Thus, amino-acid sequences were produced to gain insight into the BrrCIPK functional domains within each residue position.

We further searched for the conserved motifs in both BrrCBL and BrrCIPK proteins using the MEME program to analyze the diversity of motif compositions. A total of 15 conserved motifs designated as motif 1 to motif 15, respectively, were identified within the genes (Supplementary Figures 5, 6). These motifs may also help predict the genes' functions. Our analysis revealed that motif 10 contained the N-myristoylation motif of 12 BrrCBL proteins (Supplementary Figure 5). For the BrrCIPKs, motifs 1 and 2 were found within the PKC domain. Motif 8 in the protein–protein interaction (PPI) domain was shared by all BrrCIPKs. Motifs 7 and 11, also shared by all the BrrCIPK members, were found within the region of the NAF/FISL domain and showed relatively conserved amino acids (N, A, F, I, S, and L; Supplementary Figure 6). The amino acid residues at the 2nd, 3rd, 4th, 7th, and 10th sites of the NAF/FISL motif were rather conserved (Supplementary Figure 4B). The phylogenetic analysis together with the structure analysis presented herein may facilitate the functional annotation and study of CBLs and CIPKs in turnip.

### Chromosomal location analysis and evolutionary history of the turnip BrrCBL and BrrCIPK gene families

**Figure 3:**
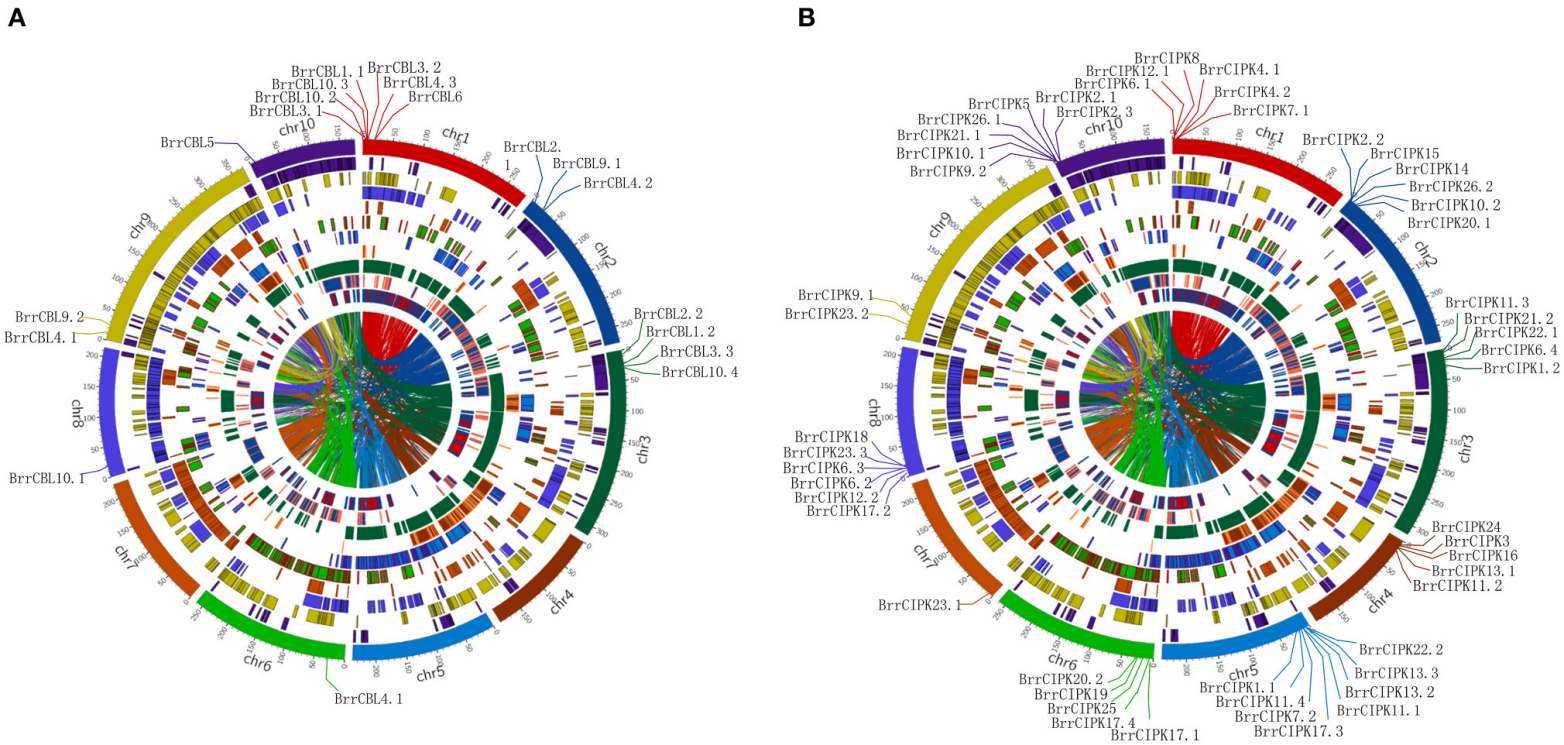
Turnip CBLs and CIPKs Chromosome distributions. Ten kinds of color in the outer circle represented 10 turnip chromosomes. Within the circle were numerous duplications between and within chromosomes. The distribution of BrrCBL and BrrCIPK on 10 chromosomes were given in the outer circle, where the numbers represent the chromosome length in 100 kb. **(A)** The distribution of BrrCBL on 10 chromosomes (Chromosome 4, 5, 7 containing no BrrCBLs). **(B)** BrrCIPKs variably distributed among all on the turnip chromosomes.

To determine the relationship between gene duplication and genetic divergence in the CBL or CIPK gene family in turnip, we investigated the chromosomal location of BrrCBL and BrrCIPK (Figure 3). All the BrrCBLs and BrrCIPKs were located at the top sections of chromosomes. Among the 10 chromosomes, chromosome 1 contained the maximum number of seven BrrCBL genes. Chromosomes 4, 5, and 7 carried no BrrCBL. Chromosomes 6, 8, and 10 each contained only one BrrCBL gene, and all other chromosomes contained 2–4 BrrCBLs (Figure 3A). Given the phylogenetic analyses and an entire genome analysis of gene duplications, we observed that six pairs of BrrCBL were putative paralogs, including five segmental duplications (*BrrCBL1.1*/*1.2, BrrCBL2.1*/*2.2, BrrCBL3.1*/*3.3, BrrCBL4.1*/*4.3*, and *BrrCBL9.1*/*9.2*) and one tandem duplication (*BrrCBL10.2*/*10.3*) (Table 3). Furthermore, we found that some paralog genes were located on different chromosomes or at different locations on the same chromosome (Figure 3B). Two pairs of *BrrCBL3.1*/*3.3* and *BrrCBL10.2*/*10.3* were located on the same chromosomes at different locations. Meanwhile, other paralogs were located on different chromosomes. Among all the genes studied, the BrrCIPK genes were found variably distributed on the turnip chromosomes. Chromosomes 5 and 9 contained the maximum and the minimum numbers, respectively, of BrrCIPK genes. A total of 17 pairs of putative BrrCIPK paralog genes were produced by segmental duplication, including 14 duplication events between chromosomes (*BrrCIPK1.1*/*1.2, BrrCIPK2.2*/*2.3, BrrCIPKV6.1*/*6.2, BrrCIPK7.1*/*7.2, BrrCIPK9.1*/*9.2, BrrCIPK10.1*/*10.2, BrrCIPK12.1*/*12.2, BrrCIPK13.2*/*13.3, BrrCIPK20.1*/*20.2, BrrCIPK21.1*/*21.2, BrrCIPK22.1*/*22.2, BrrCIPK23.1*/*23.2, BrrCIPK5*/*25*, and *BrrCIPK26.1*/*26.2*), as well as three duplication events within the same chromosome (*BrrCIPK4.1*/4*.2, BrrCIPK11.1*/*11.4*, and *BrrCIPK17.1*/*17.4*; Figure 3B, Table 3). These observations suggest the large-scale segmental duplication events involved in the expansion of BrrCBL and BrrCIPK gene families in turnip.

**Table 3:** Inference of divergence time in paralogous pairs.

**Seq 1**	**Seq 2**	**Identity (%)**	**Ks**	**Ka**	**ω**	**T(MYA)**
BrrCBL1.1	BrrCBL1.2	95.77	0.4805	0.016	0.0333	16.0167
BrrCBL2.1	BrrCBL2.2	84.03	0.5211	0.0163	0.0313	17.3700
BrrCBL3.1	BrrCBL3.3	45.66	0.3839	0.0259	0.0675	12.7967
BrrCBL4.3	BrrCBL4.1	82.81	0.3847	0.0221	0.0574	12.8233
BrrCBL9.1	BrrCBL9.2	97.18	0.2755	0.0162	0.0588	9.1833
BrrCBL10.2	BrrCBL10.3	75.5	0.1909	0.1059	0.5547	6.3633
BrrCIPK1.1	BrrCIPK1.2	89.24	0.2438	0.0336	0.1378	8.1267
BrrCIPK 2.3	BrrCIPK 2.2	79.05	0.5215	0.088	0.1687	17.3833
BrrCIPK 4.1	BrrCIPK 4.2	78.79	0.2878	0.0812	0.2821	9.5933
BrrCIPK 6.2	BrrCIPK 6.1	98.87	0.0503	0.0055	0.1093	1.6767
BrrCIPK 7.1	BrrCIPK 7.2	85.38	0.2723	0.0986	0.3621	9.0767
BrrCIPK 9.1	BrrCIPK 9.2	89.93	0.3327	0.0342	0.1028	11.0900
BrrCIPK 10.1	BrrCIPK 10.2	84.63	0.6288	0.0576	0.0916	20.9600
BrrCIPK 11.1	BrrCIPK 11.4	81.72	0.0449	0.0036	0.0802	1.4967
BrrCIPK 12.1	BrrCIPK 12.2	90.28	0.4651	0.0316	0.0679	15.5033
BrrCIPK13.3	BrrCIPK13.2	85.48	0.5135	0.0558	0.1087	17.1167
BrrCIPK17.4	BrrCIPK 17.1	96.47	0.1345	0.0509	0.3784	4.4833
BrrCIPK20.1	BrrCIPK20.2	68.12	0.4145	0.0877	0.2116	13.8167
BrrCIPK 21.1	BrrCIPK 21.2	99.28	0.0434	0.0018	0.0415	1.4467
BrrCIPK 22.1	BrrCIPK 22.2	90	0.8446	0.0591	0.0700	28.1533
BrrCIPK 23.1	BrrCIPK 23.2	89	0.5742	0.0213	0.0371	19.1400
BrrCIPK25	BrrCIPK5	74.62	0.8202	0.1181	0.1440	27.3400
BrrCIPK 26.2	BrrCIPK 26.1	86.88	0.1418	0.0622	0.4386	4.7267

*Ks, synonymous substitution rates; Ka, Nonsynonymous substitution rates; MYA, million years ago*.

We estimated the divergence time (T) of six pairs of turnip putative BrrCBL paralog proteins by measuring the synonymous (Ks) and nonsynonymous (Ka) mutation rates (Table 3). In the process, we used a divergence rate of 1.5 × 10^−8^ mutations per synonymous site per year. The estimated divergence time (T) for the turnip CBL paralogs was approximately between 6.3633 and 17.3700 million years ago (MYA), with an average duplication time of ~12.4256 MYA. Ka/ks (ω) was calculated for each pair of CBL paralog genes. The ω-values for all of the putative CBL paralogs with mean value of 0.134 were less than one. Thus, the five pairs of turnip CBL proteins were potentially under strong purifying selection pressure. However, one pair of turnip CBL genes (*BrrCBL10.2*/*BrrCBL10.3*, ω = 0.5547) attained relatively large ω-values. This result implied that the corresponding genes may have evolved rapidly from the last common ancestor. We also estimated the divergence time of 17 pairs of turnip putative CIPK paralog genes. Moreover, the estimated T for BrrCIPK paralogs was between 1.4467 and 28.1533 MYA, with an average duplication time of approximately 12.4194 MYA. Interestingly, the divergence time of three BrrCIPK paralogs (*BrrCIPK6.1*/*6.2, BrrCIPK11.1*/*11.4*, and *BrrCIPK5*/*25*) were estimated to have occurred recently (only approximately one). Schranz and Mitchell–Olds (Schranz et al., 2006) estimated the time of very early radiation of Brassicaceae at 34 MYA. Other authors proposed that the conserved linkage arrangements between *B. rapa* and *A. thaliana* diverged from a common ancestor at ~14.5–20.4 MYA (Ferguson et al., 2003). However, the divergence time of three pairs of BrrCIPK paralogs (*BrrCIPK10.1*/*10.2, BrrCIPK22.1*/*22.2*, and *BrrCIPK5*/*25*; 20.9600–28.1533 MYA) occurred precedent to the period of the origin of the *B. rapa*. The ω in all the putative BrrCIPK paralogs with mean value of 0.167 were less than one. Hence, the 17 pairs of turnip BrrCIPK genes were possibly under strong purifying selection pressure. By contrast, three pairs of BrrCIPK genes, namely, *BrrCIPK7.1*/*7.2* (ω = 0.3621), *BrrCIPK17.1*/*17.2* (ω = 0.3784), and *BrrCIPK26.1*/*26.2* (ω = 0.4386), achieved relatively large ω-values, which indicate that they may have evolved rapidly from those of the last common ancestor.

### Expression profiles of turnip BrrCBL and BrrCIPK genes at different tissues and different developmental stages

**Figure 4:**
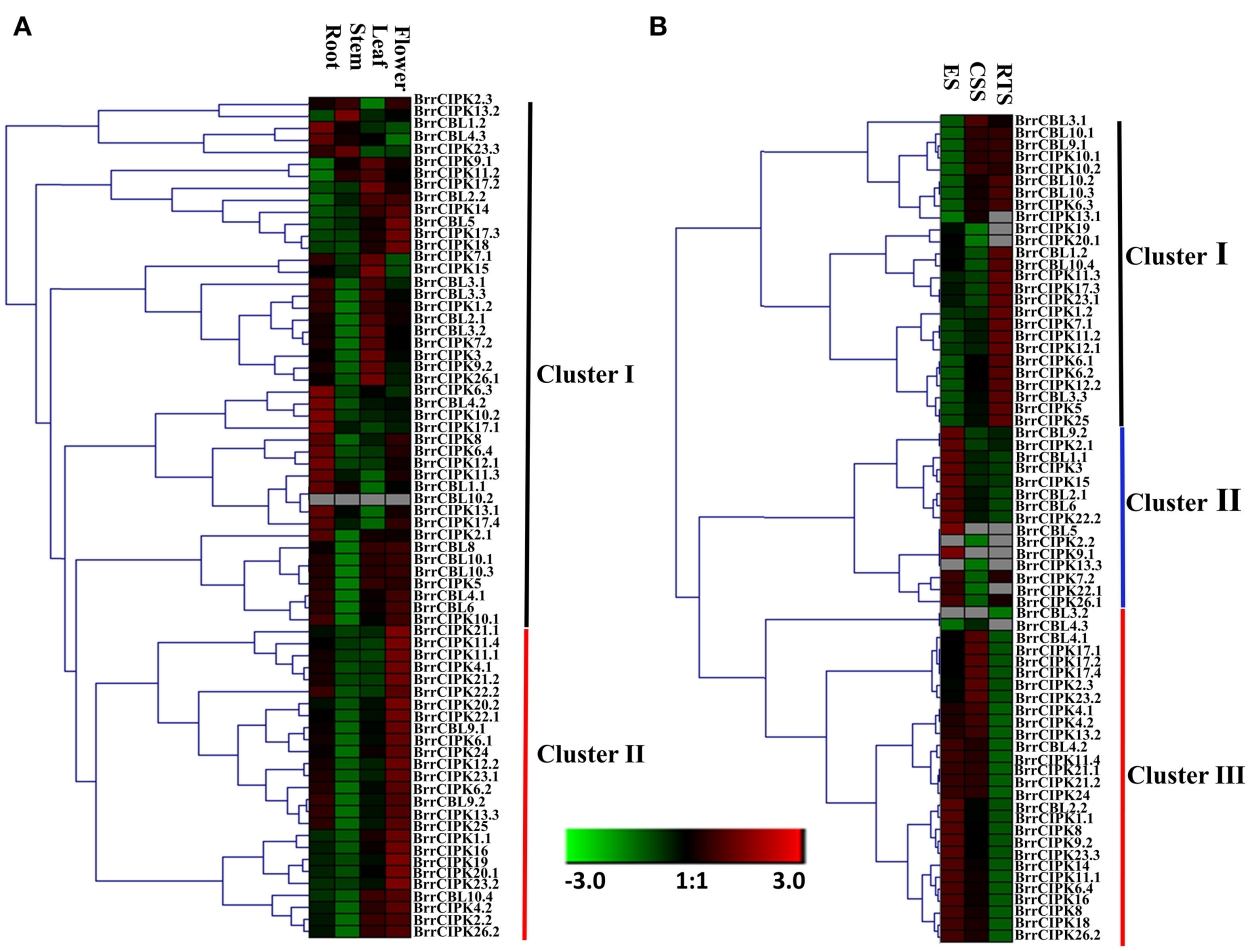
Expression profiles of turnip CBL and CIPK genes in different tissues and different development stages. **(A)** Heat map of real-time quantitative PCR (qRT-PCR) analysis of BrrCBL and BrrCIPK genes in the four tissues of roots, stems, leaves, flowers, with three biological and technical replicates. **(B)** Heat map of expression profiles (in log2-based FPKM) for BrrCBL and BrrCIPK in early stage before cortex splitting (ES), cortex splitting stage (CSS), and secondary root thickening stage (RTS). The expression levels were represented by the color bar.

We determined the spatial and temporal expression profiles of 19 BrrCBL and 51 BrrCIPK genes in different tissues of turnip (root, stem, leaf, and flower) by quantitative real-time PCR (qRT-PCR; Figure 4A). A heat map was created through the hierarchical clustering of the gene expression profiles of the BrrCBL and BrrCIPK genes. These profiles can be classified into two clusters by expression pattern. Cluster I consisted of 16 BrrCBLs and 28 BrrCIPKs with different expression patterns in different tissues. Some of these genes were highly expressed in certain tissue tested. *BrrCBL1.1*, -*1.2*, -*4.2*, and -*4.3*, as well *BrrCIPK6.3*, -*10.2*, and -*17.1*, almost showed peak transcript levels in the root. *BrrCIPK13.2* and *23.3* were highly transcribed in the stem, whereas *BrrCIPK3*, -*9.1*, -*15*, -*17.2*, and -*26.1* were highly transcribed in the leaf. These transcriptional patterns indicate that these genes may be involved in organ development and growth. By contrast, some genes, especially *BrrCBL10.2*, showed no transcript levels in all tissues. Cluster II included three BrrCBL and 23 BrrCIPK genes with high transcript abundance in the flowers. Hence, the genes in Cluster II may play important roles in flower development. Additionally, some paralogs showed opposite expression patterns. Examples include 4 out of 6 BrrCBL paralogs (*BrrCBL2.1*/*2.2, BrrCBL4.1*/*4.3, BrrCBL10.2*/*10.3*, and *BrrCBL12.1*/*12.2*) and 4 out of 17 BrrCIPK paralogs (*BrrCIPK1.1*/*1.2, BrrCIPK2.2*/*2.3, BrrCIPK17.1*/*17.4*, and *BrrCIPK26.1*/*26.2*) in different tissues (Figure 4A).

The expression levels of BrrCBL and BrrCIPK genes were reanalyzed using publicly available RNA-sequence data from three different developmental stages (Li et al., 2015). These stages include the average of the early stage before cortex splitting (ES1 and ES2), the cortex-splitting stage (CSS1 and CSS2), and the secondary root-thickening stage (RTS1 and RTS2) in turnip corresponding to ES, CSS, and RTS, respectively (Figure 4B). Likewise, the transcripts of the BrrCBL and BrrCIPK genes can be detected in different developmental stages and can be divided into three clusters by expression pattern. The eight BrrCBL and 18 BrrCIPK genes were assigned to Cluster I, except *BrrCIPK13.1*, -*19*, and -*20.1*, which were relative highly expressed in the secondary RTS. Five BrrCBL and 10 BrrCIPK genes were found in Cluster II, and they were almost exclusively expressed in the early stage before cortex splitting (ES), except for *BrrCIPK2.2* and *BrrCIPK13.3*. Among the Cluster III genes, 6 BrrCBL and 23 BrrCIPK genes were highly expressed in the early stage before cortex splitting (ES) and CSS. By contrast, the expression of *BrrCBL3.2* was not detected and *BrrCBL4.3* was expressed at a very low level. These results show that some of the genes were modestly expressed in developmental stages. Meanwhile, some genes were specifically and abundantly expressed in certain developmental stages. Therefore, such genes may play important role during developmental stages. We also found that a subset of paralogous genes were differentially expressed in the three developmental stages (Figure 4B). *BrrCBL9.1* was expressed abundantly in the CSS and RTS stages, whereas *BrrCBL9.2* was specifically expressed in the ES. The expression patterns of two BrrCBL paralogous pairs (*BrrCBL1.1*/*1.2* and *BrrCBL4.1*/*4.3*) and six BrrCIPK paralogous pairs (*BrrCIPK1.1*/*1.2, BrrCIPK2.2*/*2.3, BrrCIPK7.1*/*7.2, BrrCIPK13.2*/*13.3, BrrCIPK7.1*/*7.2*, and *BrrCIPK23.1*/*23.2*) also diverged dramatically in developmental stages. Overall, the expression patterns of BrrCBL and BrrCIPK genes at different tissues and different developmental stages may indicate the functional divergence between paralogs.

### Expression profiles of BrrCBL and BrrCIPK genes during stress treatment

**Figure 5:**
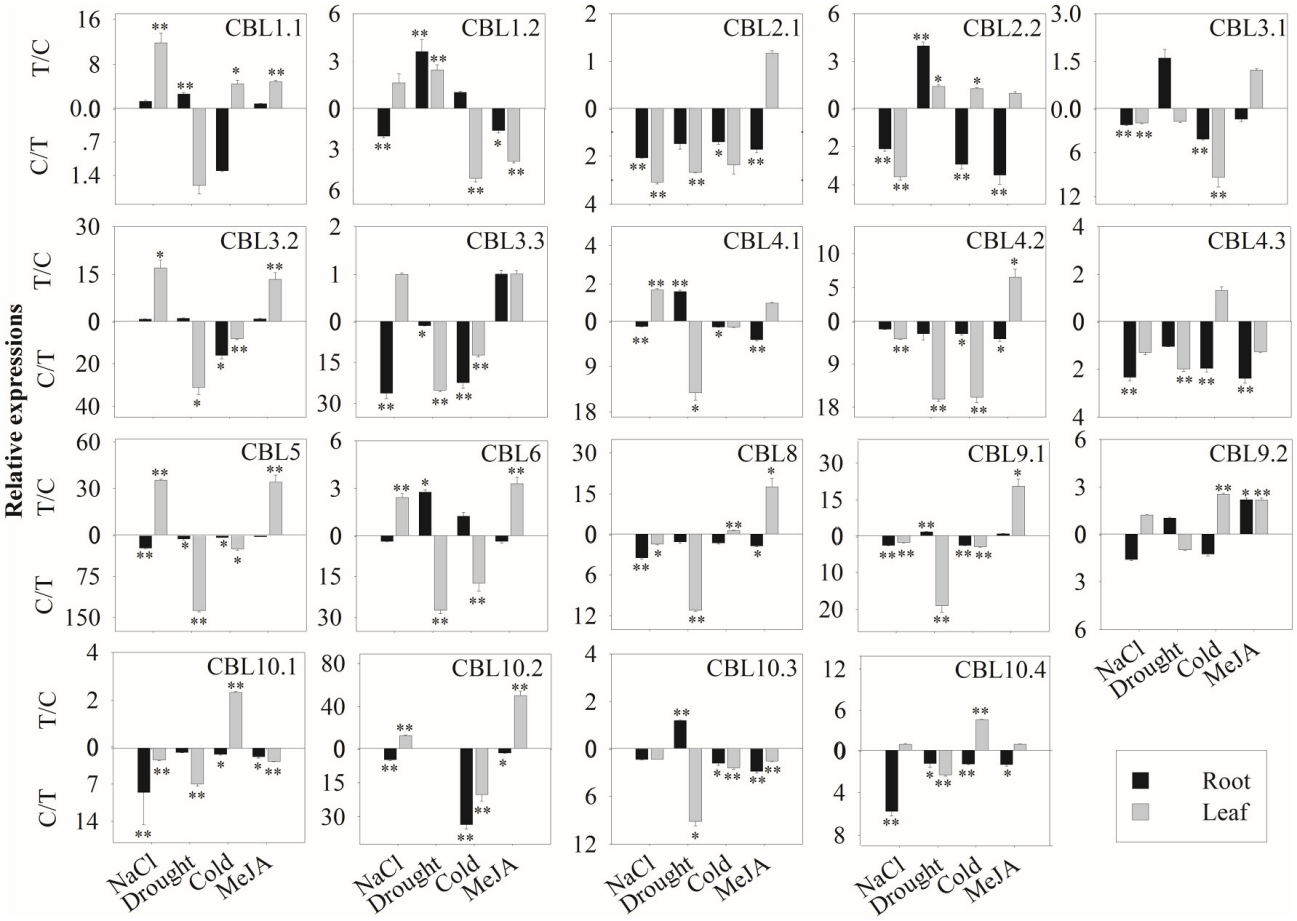
Expression analysis of BrrCBL in leaves and roots responding to various treatments, including NaCl (100 mM), drought, cold (4°C), and MeJA (1 mM). Two time points (0 and 3 h) were used to detect the gene expressions, with three biological and technical replicates. Error bars represented standard errors of repeats. The stress treatments with significant change of expression level compared to the controls with *P* < 0.05 and *P* < 0.01 were marked by “^*^” and “^**^,” respectively.

We then further investigated the expression profiles of BrrCBL and BrrCIPK genes under stress conditions. We also evaluated the possible functional divergence of paralog genes. To achieve these goals, we conducted qRT-PCR analysis. The transcript level of the *BrrCBL1.1* gene showed the most significant increases among those of other genes after various stress treatments (Figure 5). By contrast, *BrrCBL2.1*, -*4.2*, and -*4.3* expression was remarkably downregulated in four stress treatments. Under NaCl treatment, the *BrrCBL1.1*, -*3.2*, -*4.1*, -*5*, -*6*, and -*10.2* transcript levels significantly increased. Meanwhile, *BrrCBL1.2*, -*3.3*, -*9.2*, and -*10.4* expression was still slightly upregulated after NaCl treatment. Under the drought treatment, *BrrCBL1.1*, -*1.2*, -*2.2*, -*4.1*, -*6*, -*9.1*, and -*10.3* expression significantly induced. In addition, cold stress significantly upregulated the expression levels of *BrrCBL1.1*, -*2.2*, -*8*, -*9.2*, -*10.1*, and -*10.4*. In response to MeJA treatment, *BrrCBL1.1*, -*3.2*, -*5*, -*6*, -*8*, -*9.1*, -*9.2*, and -*10.2* were highly upregulated. Additionally, we investigated the expression profiles of BrrCBL paralog gene pairs and found that they exhibited different expression patterns under different stress conditions. *BrrCBL9.1* expression was downregulated, whereas *BrrCBL9.2* expression was upregulated by NaCl and cold treatments. The results suggest the functional divergence of the BrrCBL paralog pairs.

**Figure 6:**
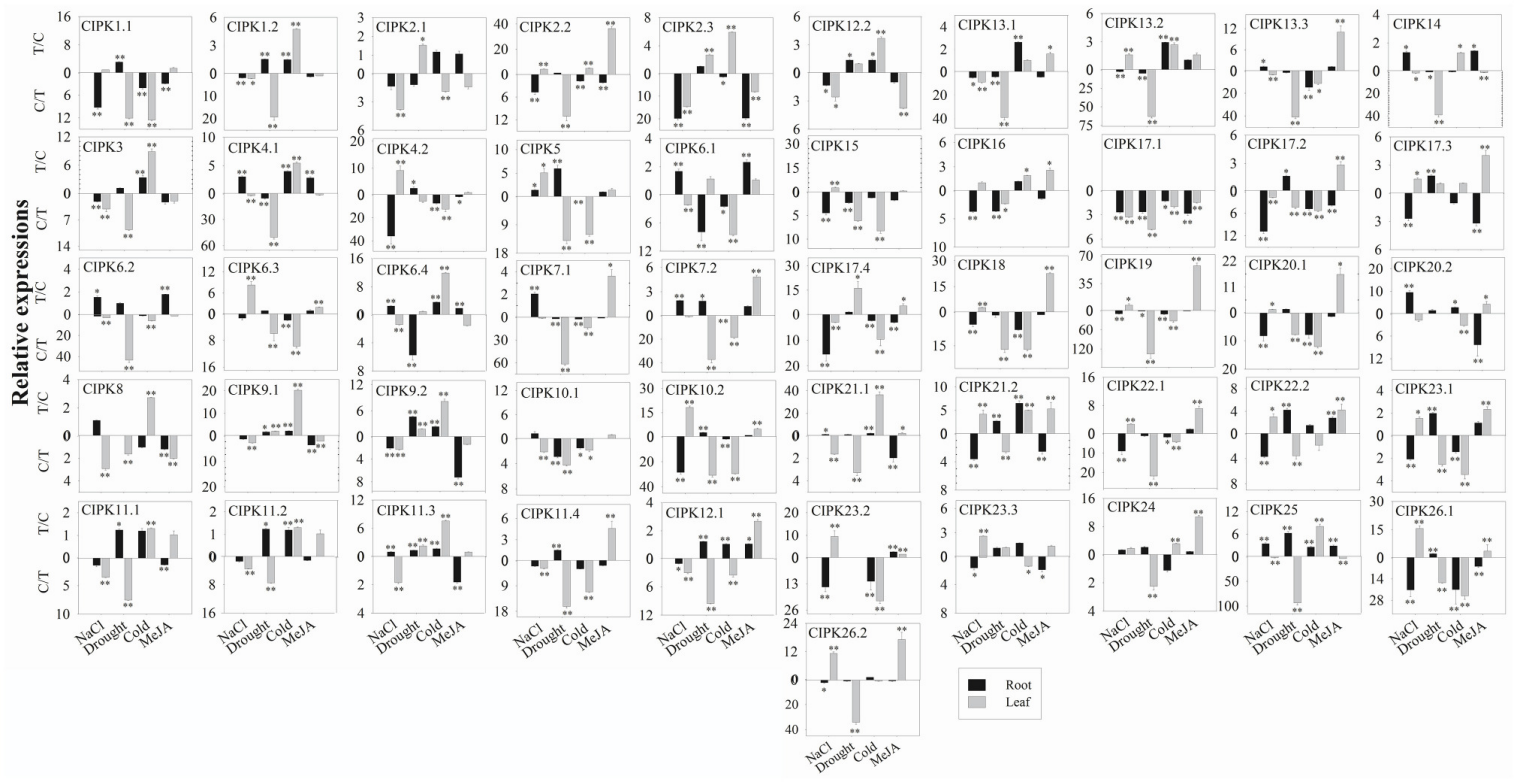
Expression analysis of BrrCIPK in leaves and roots responding to various treatments, including NaCl (100 mM), drought, cold (4°C), and MeJA (1 mM). Two time points (0 and 3 h) were used to detect the gene expressions, with three biological and technical replicates. Error bars represented standard errors of repeats. The stress treatments with significant change of expression level compared to the controls with *P* < 0.05 and *P* < 0.01 were marked by “^*^” and “^**^,” respectively.

For the 51 BrrCIPK genes assayed under four stress treatments, the most striking induction was observed for 34 BrrCIPK genes after MeJA treatment, followed by 30 BrrCIPK genes after NaCl treatment. Among these genes, *BrrCIPK2.2*, -*3.2*, -*5*, -*8*, -*9.1*, -*10.2*, -*18*, -*19*, and -*20.1* exhibited remarkable MeJA-induced upregulation. *BrrCIPK10.2*, -*20.2*, -*23.2*, and -*26.1* also exhibited remarkable NaCl-induced upregulation. Drought treatment induced the expression of 24 BrrCIPKs as that under cold treatment. Strikingly, *BrrCIPK17.4* was markedly upregulated in response to drought. The transcript levels of *BrrCIPK9.1* and *BrrCIPK21.1* showed remarkable increases after cold treatment. Conversely, the transcript levels of *BrrCIPK17.1* gene were significantly decreased under the four treatments (Figure 6). Several paralog gene pairs (such as *BrrCIPK1.1*/*1.2, BrrCIPK4.1*/*4.2*, and *BrrCIPK10.1*/*10.2*) demonstrated divergent expression patterns, that is, one gene is induced, whereas the other is suppressed by the treatment. The results reveal that the BrrCIPK paralogs exhibit distinct expression patterns under different stress conditions. This occurrence also suggests the functional divergence of the BrrCIPK-duplicated genes.

According to previous reports, CBL–CIPK complexes play highly important roles in response to external stimuli (Manik et al., 2015; Sun et al., 2015). We wish to determine whether the same stress responses of BrrCBL and BrrCIPK transcripts can interact with each other. To investigate this aspect in the turnip expression profiles, we performed coexpression analysis, which can predict the interaction of the BrrCBL–BrrCIPK complex in response to stress. A number of BrrCBLs and BrrCIPKs were upregulated and downregulated in response to one or more stress treatments (Supplementary Figure 7). For example, the transcript abundances of *BrrCBL1.1*, -*8*, -*9.2*, and -*10.4* as well as *BrrCIPK2.3*, -*8*, -*14*, and -*24* were increased by cold. We therefore speculate that potential interactions may exist between these BrrCBL and BrrCIPK genes.

### Protein interaction analysis between BrrCBLs and BrrCIPKs

**Figure 7:**
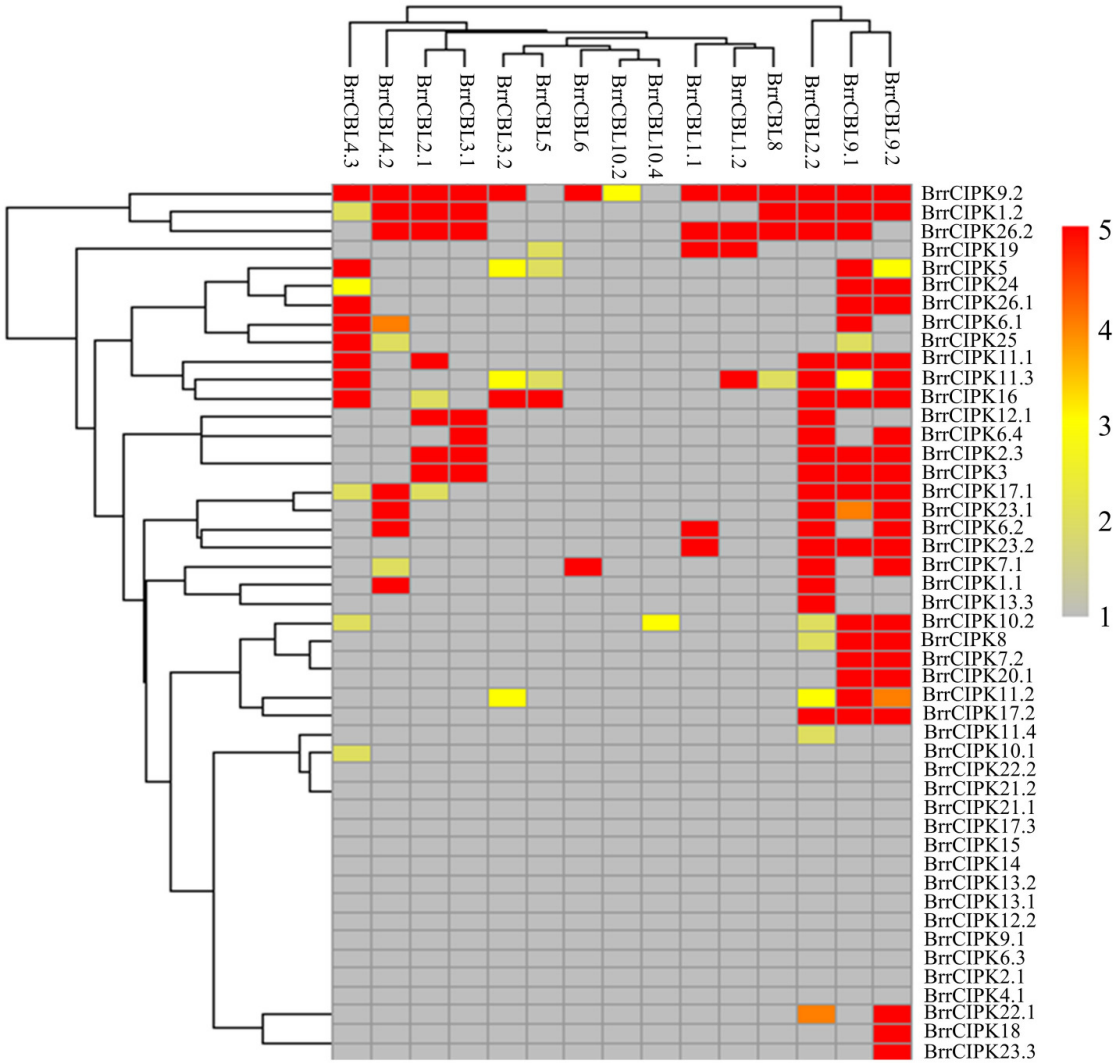
Heat map summarizing yeast two-hybrid (Y2H) results for all interactions between turnip CBL and CIPK proteins. Interaction strength was inferred by serial growth dilutions on selective media lacking one or two auxotrophic markers and summarized qualitatively by heat map. Red boxes indicate vigorous growth on -LTHA plates; Orange boxes indicate weaker growth on -LTHA plates; Yellow boxes indicate robust growth on -LTH plates but no growth on -LTHA plates; Pale yellow boxes indicate weak growth on -LTH plates, but each CBL-CIPK interaction conferred better growth than the empty vector control; Gray boxes indicate only growth on -LT plates. The phylogenetic relationships of both BrrCBLs and BrrCIPKs family were inferred by cladogram.

**Figure 8:**
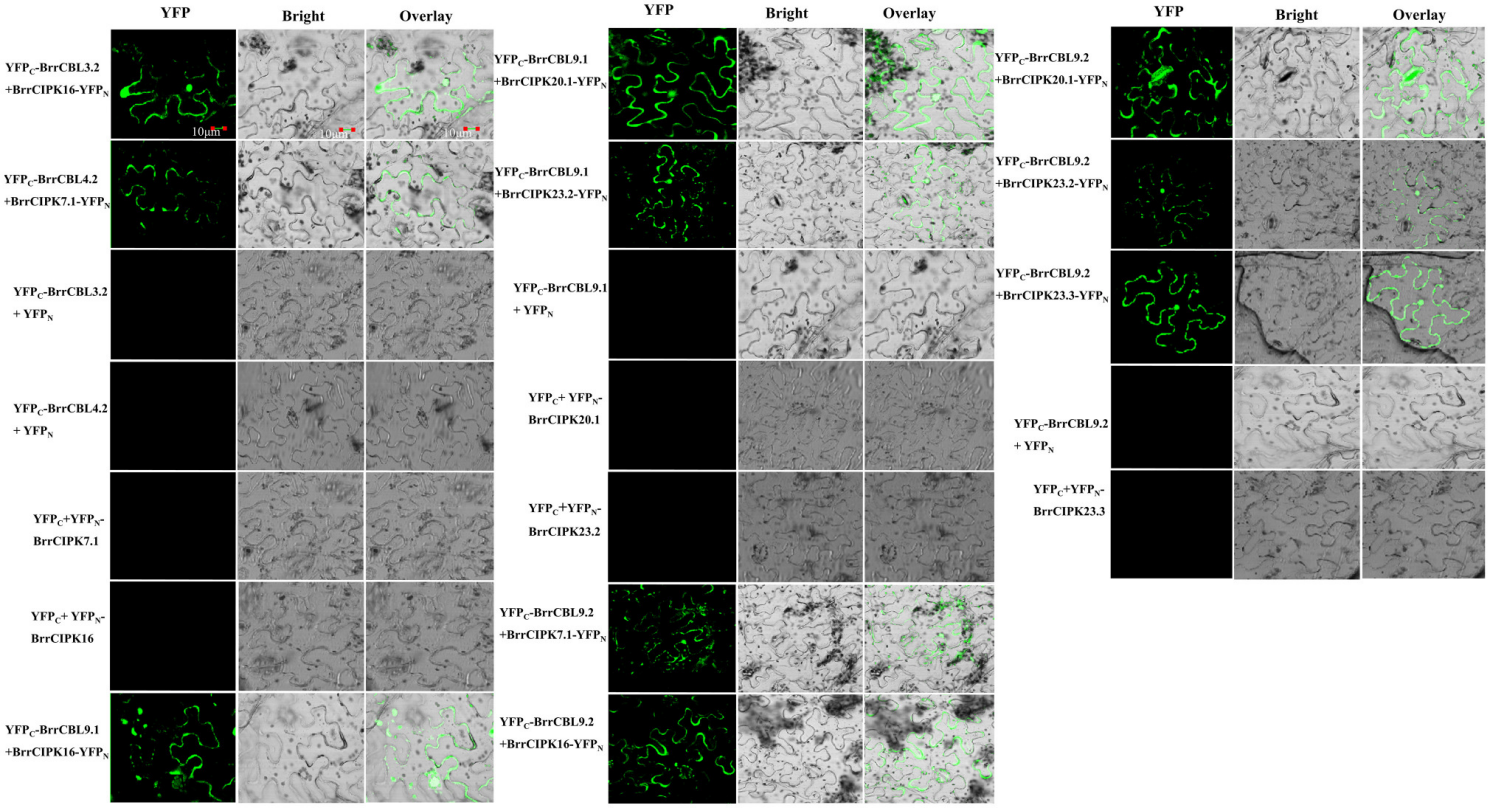
The bimolecular fluorescence complementation (BiFC) experiments in *N. benthamiana* leaf cells. The CDS regions of BrrCBL and BrrCIPK genes were sub-cloned into 35s-SPYCE and 35s-SPYNE vectors, respectively. Bar = 10 μm.

To further elucidate the interaction preferences between CBLs and CIPKs in turnip, we used a yeast two-hybrid (Y2H) system. Up to 15 BrrCBL and 47 BrrCIPK genes were cloned and used in Y2H assays. In the current study, we demonstrated 166 interactions between BrrCBLs and BrrCIPKs; the interactions displayed varying strengths and specificities (Figure 7). Notably, BrrCBL9.1 and -9.2 were paralog proteins and showed strong interactions with 22 and 25 BrrCIPKs, respectively. Some specific BrrCIPKs were identified, and these genes interacted specifically with either of the others. BrrCBL9.1 specifically interacted with BrrCIPK6.1, -25, and -26.2, whereas BrrCBL9.2 interacted specifically with BrrCIPK6.2, -6.4, -7.1, -18, -22.1, and -23.3. Thus, BrrCBL9.1 and -9.2 were involved in more interactions with BrrCIPKs than those of the other BrrCBLs. However, the two genes displayed a fairly different interaction with its paralogs, and hence may be appropriate for further in-depth research. Overall, this type of interaction analysis enabled the deduction of potential gene functions to guide future functional studies. We then selected 10 of the interactions (BrrCBL3.2-BrrCIPK16, BrrCBL4.2-BrrCIPK7.1, BrrCBL9.1-BrrCIPK16, -20.1 and -23.2, BrrCBL9.2-BrrCIPK7.1, -16, -20.1, -23.2, -23.3) detected between BrrCBL and BrrCIPK proteins using the Y2H assay for further analyses using the BiFC method. We found that green fluorescence signals were observed when BrrCBLs and BrrCIPKs were co-expressed in epidermal cells of tobacco leaves (Figure 8). It seemed that the fluorescent signal were consistent with the localization signal of corresponding BrrCBL proteins (Supplementary Figure 1). In contrast, in controls in which BrrCBL3.2, -4.2, -9.1, or -9.2 was expressed together with YFP_N_ only, as well BrrCIPK7.1, -16, -20.1, -23.2, or -23.3 with YFP_C_ only, no signal of reconstructed YFP appeared.

### Functional analysis of *BrrCBL9.1* and *BrrCBL9.2* under salt stress

**Figure 9:**
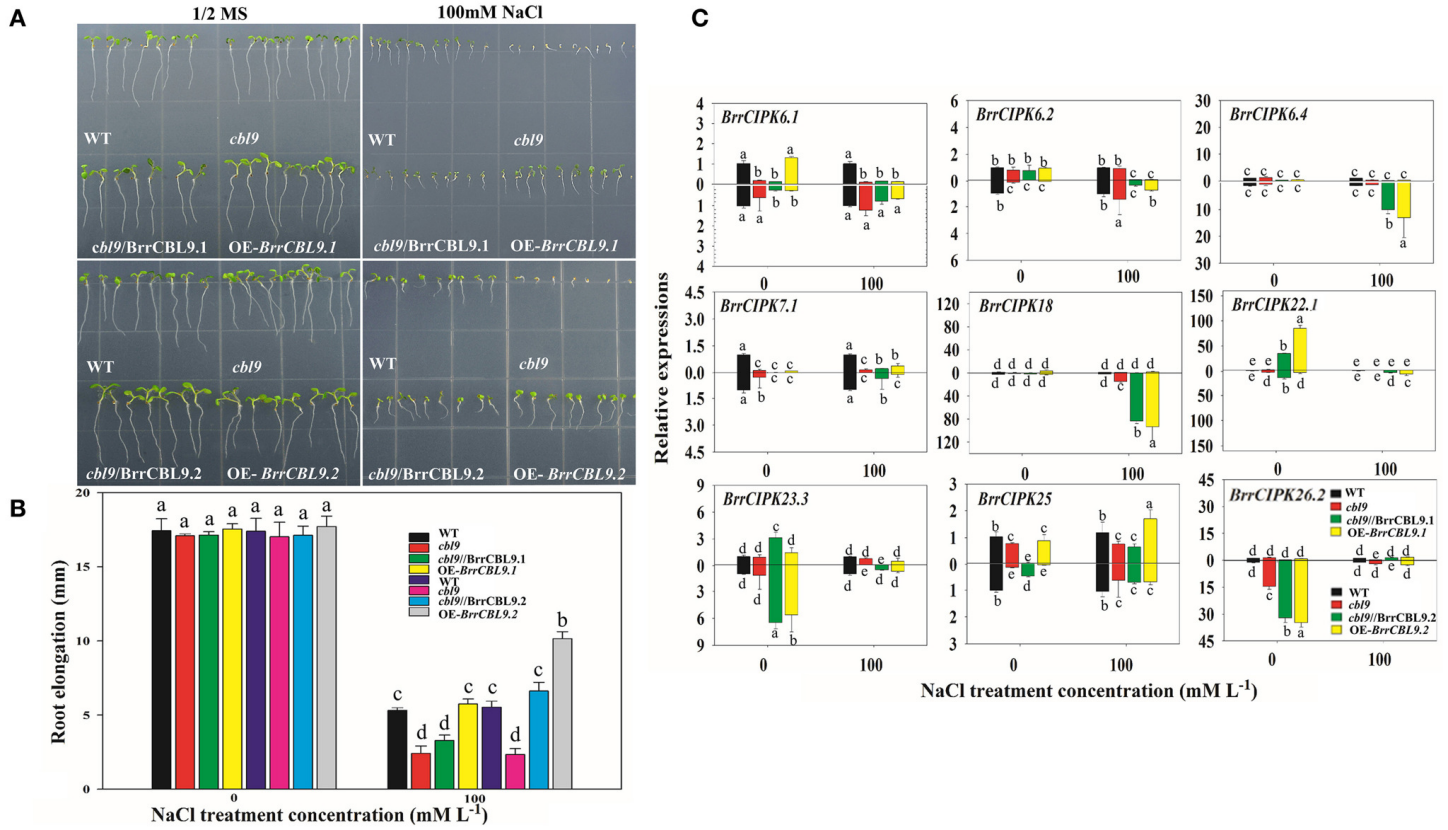
Overexpression of *BrrCBL9.1* and -*9.2* in *Arabidopsis* and functional complementation of *Arabidopsis cbl9* mutant by *BrrCBL9.1* and -*9.2* gene. **(A)** The plants grown on control (1/2MS medium) and stress condition (1/2MS + 100 mM NaCl), respectively. **(B)** Statistical analysis of the changing root length between 1/2MS control and salt stress condition. **(C)** Expression analysis of corresponding BrrCIPK genes specially interacted with BrrCBL9.1 and 9.2 in WT, *cbl9* mutant, complemented, and overexpressing plants responding to NaCl (100 mM). Two time points (0 and 3 h) were used to detect the gene expressions, with three biological and technical replicates. Error bar denoted standard deviation, and different letters within a column indicate a significant difference (*P* < 0.05; Tukey's test).

The *AtCBL9* gene in *Arabidopsis* possesses two counterparts, namely, *BrrCBL9.1*, and -*9.2*, in turnip. The differences between these genes were discovered through expression and interaction profiles. Previous studies revealed that the *Arabidopsis cbl9* mutant is hypersensitive to ABA, salt, drought, and glucose stress (Pandey et al., 2004). To dissect the biological function of turnip *CBL9.1* and -*9.2*, we overexpressed each gene in *Arabidopsis* using a CaMV 35S promoter (OE-*BrrCBL9.1* and OE-*BrrCBL9.2*, respectively). To examine whether *BrrCBL9.1* and -*9.2* can rescue the *cbl9* mutant phenotype, we transformed a *cbl9* mutant plant with *BrrCBL9.1* and -*9.2* using the CaMV 35S promoter (*cbl9*/BrrCBL9.1 and *cbl9*/BrrCBL9.2, respectively). Seeds from wild-type (WT), *cbl9* mutant, complemented, and overexpressing plants were germinated on 1/2 Murashige and Skoog (MS) and 1/2 MS with 100 mM NaCl (Figures 9A,B). On the 1/2 MS medium plates, the WT, *cbl9* mutant, complemented, and overexpressing lines displayed relatively similar growth phenotypes. The latter three plants showed no significant difference in primary root length from the WT. After 100 mM NaCl treatment, no significance difference in the primary roots was observed between *cbl9* and *cbl9*/BrrCBL9.1, as well as between the OE-*BrrCBL9.1* and WT under salt-stress conditions. However, the primary roots of *cbl9*/BrrCBL9.2 were restored to WT background levels, and the primary roots of OE-*BrrCBL9.2* were much longer than those of WT plants. Therefore, our data indicate that the *BrrCBL9.2* gene can promote root elongation in both transgenic and complementary *Arabidopsis* under salt stress. We also detected the expression level of corresponding BrrCIPK genes specially interacted with BrrCBL9.1 and -9.2 in complemented and overexpressing transgenic plants (Figure 9C). As a result, *BrrCIPK6.4* and -*18* showed the most significant increase in complemented and overexpressing transgenic *Arabidopsis* of *BrrCBL9.2* under NaCl treatment, but *BrrCBL9.1* does not affect. This indicated that the interaction between BrrCBL9.2 and BrrCIPK6.4 and -18 may play a role in salt responses.

## Discussion

The signaling system composed of CBLs and CIPKs is a key regulatory node in various stress signaling pathways in plants. Previously, CBL and CIPK members were comprehensively analyzed in many species, such as *P. patens* (four CBLs and seven CIPKs), *S. moellendorffii* (four CBLs and five CIPKs), *Arabidopsis* (10 CBLs and 26 CIPKs) (Luan et al., 2002; Kolukisaoglu et al., 2004; Zhang et al., 2014), *O. sativa* (10 CBLs and 31 CIPKs) (Kolukisaoglu et al., 2004), *Populus trichocarpa* (10 CBLs and 27 CIPKs) (Yu et al., 2007; Zhang et al., 2008), *Z. mays* (Chen X. F. et al., 2011) (eight CBLs and 43 CIPKs), *B. napus* L. (seven CBLs and 23 CIPKs) (Zhang et al., 2014), and *S. melongena* L. (five CBLs and 15 CIPKs) (Li et al., 2016). The studies suggested that the CBLs and CIPKs developed during early plant evolution. However, no CBL and CIPK gene has been found in turnip (*B. rapa* var. *rapa*). In our study, a genome-wide database search based on conserved domains and sequence similarities to known *Arabidopsis* CBLs and CIPKs revealed 19 BrrCBL and 51 BrrCIPK genes in the turnip genome (Tables 1,2). The phylogenetic analysis indicated that BrrCIPK family can be divided into intron-rich and intron-less clades, similar to those in *Arabidopsis* and rice (Kolukisaoglu et al., 2004; Chen X. F. et al., 2011; Mao et al., 2016). And an alternative mRNA splicing of *OsCIPK3* and *OsCIPK24* were detected, as observed in *AtCIPK3* and *AtCIPK9* (Kolukisaoglu et al., 2004; Mao et al., 2016). Moreover, BrrCIPK harbored 7 to 15 introns and exhibited a high degree of intron conservation with their relative *Arabidopsis* gene family members (Kolukisaoglu et al., 2004). Thus, we speculated that the clustering of BrrCIPK was possibly due to evolutionarily intron retention and alternative splicing mechanism in turnip. This structural differences in BrrCIPK indicated they may be involved in different stress response. Previous studies revealed that *B. rapa* underwent a polyploidization event, such as the γ triplication (135 MYA), β (90–100 MYA), and α (24–40 MYA) duplications (Wang and Kole, 2015). These three polyploidization events occurred in the evolutionary history of turnip. These events were followed by chromosomal reduction and rearrangement as well as numerous gene losses. As a result, highly complex gene families were obtained. In our study, 19 BrrCBL and 51 BrrCIPK members were always clustered closely with AtCBL and AtCIPK orthologs. However, *CBL7* presented in *Arabidopsis* (Kolukisaoglu et al., 2004) but was absent in turnip mainly because of gene loss. Thus, we conclude that some of the CBL family members were conserved, whereas others were lost after the divergence. Many CBL and CIPK genes in *Arabidopsis* also held two or more counterparts in turnip. This observation suggests that the expansion of the CBL and CIPK families in turnip may be caused by genome duplication processes, including multiple segmental duplication, tandem duplication, transposition events, and entire-genome duplication (Cannon et al., 2004; Flagel and Wendel, 2009). We found evidence for five (26.3%) segmental duplication and one (0.5%) tandem duplication in 19 BrrCBLs and 17 (33.3%) segmental duplication in 51 BrrCIPK genes (Figure 3, Table 3). This finding indicates that segmental duplication predominantly contributed to the expansion of both BrrCBL and BrrCIPK in turnip. The increase in the number of CBL and CIPK genes during plant evolution was likely related to the functional evolution of the CBL–CIPK signaling system (Weinl and Kudla, 2009).

The polyploidization event also caused structural and functional domain diversification. As first found, *BrrCBL3.3* contained the largest number of introns (15 introns) in its coding regions relative to those in *AtCBL3* (Mahajan et al., 2006), *OsCBL3* (Kanwar et al., 2014), *ZmCBL3* (Zhang et al., 2016), and *PsCBL3* (Mahajan et al., 2006). The large number of introns may have been produced by structural diversification (Figure 1). Likewise, BrrCBL3.3 attained the highest molecular weight (MW) (50.486 kDa; Table 1). By contrast, CBL3 proteins in other plants were relatively conserved in size (MW ranged from 23 to 29 kDa) (Shen et al., 2014; Zhang et al., 2014). BrrCBL proteins possessed variable N-terminal, which play essential roles in the localization of CBL proteins (Supplementary Figure 3). The two segmental duplication pairs (BrrCBL2.1/2.2 and BrrCBL3.1/3.2) differed in the N-terminal. Confocal fluorescence microscopy analysis showed that BrrCBL2.1 and -3.1 localized in the plasma membrane. By contrast, their segmental duplication partners BrrCBL2.2 and -3.2 localized in the plasma membrane and nuclei (Supplementary Figure 1). This result differed from that of the vacuolar membrane-localized proteins AtCBL2 and -3 in *Arabidopsis* (Batistic et al., 2008). However, both BrrCBL1.1/1.2 and BrrCBL9.1/9.2 (harboring the same myristoylation motif in the N-terminal) localized in the plasma membrane and nuclei (Supplementary Figure 1). AtCBL1 and -9 localized to the plasma membrane (Batistic et al., 2010). Even the intronless BrrCIPKs were highly conserved in the intron phase. Variations in intron lengths also existed in the BrrCIPKs (Figure 2). The longest intron length was found in *BrrCIPK4.1* (5.37 kb) relative to those of *AtCIPK4* (Li et al., 2009; Mao et al., 2016) and *TaCIPK4* (Sun et al., 2015). Previous studies showed that the NAF/FISL domain itself was sufficient to mediate CBL interaction (Albrecht et al., 2001; Guo et al., 2001). Even at the conserved sites of this motif, variations existed in the turnip CIPK proteins (Additional file 4). The second amino-acid residue of the NAF/FISL motif in BrrCIPK7.1, -7.2, -4.1, and -4.2 was T (threonine) instead of N (asparagine), similar to that in BnaCIPK7 in canola (Zhang et al., 2014), AtCIPK4, and AtCIPK7 in *Arabidopsis* (Li et al., 2009). This difference in amino-acid residue of a BrrCIPK protein may influence its ability to interact with BrrCBLs. Changes in structure may result in the functional divergence between paralog genes. Polyploidization events that occurred in *B. rapa* played important roles in the evolution of the plant species. This even afforded the plant with the ability to be diversified and to respond to changing habitats. The genes generated from polyploidization events developed new functions (Wang and Kole, 2015).

The large size of these two gene family members in turnip indicated their importance in the control of turnip-specific processes. In our study, the expression profiles of 19 CBLs and 51 CIPKs in different tissues and different developmental stages revealed that the majority of BrrCBL and BrrCIPK genes are expressed at different levels in all organs and at all developmental stages (Figure 4). By contrast, the expression patterns of some paralogous pairs, as such *BrrCBL9.1*/*9.2*, differed dramatically. Differences were also noted in the tandemly duplicated pair (*OsCIPK12*/*30*) and segmentally duplicated pairs (*OsCIPK6*/*27, OsCIPK1*/*17*, and *OsCIPK3*/*31*) in rice. Meanwhile, considering all the results for abiotic stresses and phytohormones, we noted that the transcript levels of all the tested BrrCBL and BrrCIPK genes either increased or decreased in response to many of the treatments (Figures 5, 6). Conversely, the responses of *BrrCBL9.2* were significantly upregulated, whereas *BrrCBL9.1* was downregulated by NaCl stress. Similar to paralog pairs *OsCIPK3* and *OsCIPK31* in rice and *ZmCIPK8* and -*31* in maize under stress treatments (Chen X. et al., 2011; Kanwar et al., 2014). Hence, this type of differential expression in paralog genes reflects that CBL and CIPK genes evolved some new functions for turnip adaptation to the adverse environmental factors in the Qinghai–Tibet Plateau. Moreover, the CBL and CIPK contribute to plant stress-response mechanisms usually by forming a CBL–CIPK signaling component (Manik et al., 2015; Mao et al., 2016). Among the interactions, we found that the paralog pair BrrCBL9.1 and -9.2 can interact with 22 and 25 BrrCIPKs, respectively (Figure 7). They had the common and specific interactors with BrrCIPKs. In this regard, they may serve functional roles in different signaling pathways and choose different interacting partners to propagate signals downstream. Along with the differences between BrrCBL9.1 and -9.2 in expression patterns and interactions, we speculated that the two genes serve dissimilar functions.

The functional divergence of homologous genes may play a vital role in a plant's adaptation to adverse environments. In *Arabidopsis, AtCBL1* functions as a positive regulator of salt and drought responses and as a negative regulator of cold responses in plants (Cheong et al., 2003). Meanwhile, *AtCBL9* acts as a negative regulator of ABA signaling and is involved in ABA biosynthesis under stress (Pandey et al., 2004). Similar findings were obtained for *Arabidopsis CIPK8* (Hu et al., 2009) and *CIPK24* (Liu et al., 2000). Our transgenic/complement *Arabidopsis* assay indicated that *BrrCBL9.2* play roles in salt responses. This result suggests that the gene is not functionally redundant (Figure 9). Previous studies of *CBL9* in other species demonstrated that overexpressing *ZmCBL9* enhances the resistance or tolerance to ABA, glucose, salt, and osmotic stress. The studies also showed that the process complements the hypersensitive phenotype of the *Arabidopsis cbl9* mutant in response to ABA and abiotic stress (Pandey et al., 2004; Zhang et al., 2016). Overexpressing *Thellungiella halophila ThCBL9* increases salt and osmotic stress tolerance in transgenic *Arabidopsis* (Sun et al., 2008). These results indicated that *BrrCBL9.2* may play a similar molecular function to those of *AtCBL9, ZmCBL9*, and *ThCBL9* in salt response. Thus, an evolutionary conserved function of *BrrCBL9.2* genes may exist between dicots and monocots. However, *BrrCBL9.1* may serve some new functions in other stress responses. Thus, the functional divergence of the paralog pair *BrrCBL9.1* and -*9.2* may play a prominent role in turnip's adaptation to the environment of the Qinghai–Tibet Plateau.

In conclusion, we provided a systematic genome-wide analysis of the CBL and CIPK gene families in turnip. Up to 19 and 51 members of the BrrCBL and BrrCIPK genes were identified, respectively. Phylogenetic and chromosomal distribution analyses revealed that the main reason for the expansion of these two gene families was a segmental duplication event. Different expression patterns showed that the BrrCBL and BrrCIPK involved various stresses and some paralog genes functionally diverged during long-term evolution. Y2H assay demonstrated the interaction complex between BrrCBLs and BrrCIPKs and the different interactions between some paralog proteins. Further functional analysis suggested that *BrrCBL9.2* was upregulated by salt stress. Overall, this study provides insight into the characterization of CBLs and CIPKs in turnip. These insights then contributes to the understanding of the mechanisms and functions of CBL–CIPK complexes and offers basis for selecting appropriate genes for the in-depth functional studies of CBL–CIPK in turnip.

## Author contributions

Designed the experiments: YoY, YuY, and XY. Performed the experiments: XY, QC, and NX. Analyzed the data: YuY and XY. Contributed reagents/materials/analysis tools: XY, QC, NX, and QW. Wrote the paper: XY.

### Conflict of interest statement

The authors declare that the research was conducted in the absence of any commercial or financial relationships that could be construed as a potential conflict of interest.

## Supplementary material

Supplementary Table 1 Forward and reverse primers used in gene cloning, expression analysis, Y2H and BiFC vector constructions.

Supplementary Figure 1 Subcellular localization of 15 *35S*: *BrrCBL*-GFP fusion protein in *N. benthamiana* leaf cells. Bar = 10 μm.

Supplementary Figure 2 Phylogenetic relationships of BrrCBL and BrrCIPK proteins in *Arabidopsis* and *Brassica rapa* var. *rapa*. **(A)** Phylogenetic relationships of BrrCBL proteins in *Arabidopsis* and *Brassica rapa* var. *rapa*. **(B)** Phylogenetic relationships of BrrCIPK proteins in *Arabidopsis* and *Brassica rapa* var. *rapa*. The protein sequences were aligned using the MAFFT version 7 program, and phylogenetic trees were constructed using the MEGA 5.0 software with the neighbour-joining method and the 1,000 bootstrap test replicates.

Supplementary Figure 3 Multiple sequence alignments of the amino acids of 19 BrrCBLs and 10 *Arabidopsis* CBLs. The myristoylation and palmitoylation site was the red rectangle. The four EF-hand conserved domains were all indicated with *straight lines*.

Supplementary Figure 4 Multiple sequence alignments of the amino acids of 51 turnip BrrCIPKs and *Arabidopsis* CIPK24. **(A)** CIPK proteins consist of a conserved N-terminal kinase domain, and a C-terminal regulatory domain. The activation loop was denoted by *straight lines*. The conserved NAF/FISL motif within the rather divergent C-terminal regulatory domain was denoted by dashed lines. The protein-phosphatase interaction (PPI) motif within the C-terminus of these kinases was marked by dots above the sequences. **(B)** An MEME analysis of the NAF/FISL motif in 51 BrrCIPKs.

Supplementary Figure 5 An MEME analysis of motif compositions of BrrCBLs. A total of 15 conserved motifs designated as motif 1 to motif 15 were identified within the genes.

Supplementary Figure 6 An MEME analysis of motif compositions of BrrCIPKs. A total of 15 conserved motifs designated as motif 1 to motif 15 were identified within the genes.

Supplementary Figure 7 The co-expression analysis among BrrCBLs and BrrCIPKs under NaCl, drought, cold, and MeJA treatment, respectively.
