# Supplementary material for: Genome-Wide Identification and Functional Analysis of the Calcineurin B-like Protein and Calcineurin B-like Protein-Interacting Protein Kinase Gene Families in Turnip (Brassica rapa var. rapa)
Source: Front Plant Sci. 2017 Jul 7;8:1191. doi: 10.3389/fpls.2017.01191 (PMC5500646; doi:10.3389/fpls.2017.01191)
Supplement: Supplementary Table 1 — Forward and reverse primers used in gene cloning, expression analysis, Y2H and BiFC vector constructions. [file Table1.DOCX]

**Supplementary Table1** **Forward and reverse primers used in gene cloning, expression analysis, Y2H and BiFC vector constructions.**

| Gene | Cloning | Expression | Y2H | BiFC |
| --- | --- | --- | --- | --- |
| BrrCBL1.1 | F: ATGGGCTGCTTCCACTCAA  R: TCATGTGGCAATCTCATCG | F: TTCAAGAGCAGGAAGAGGGAG  R: GTGGAACACATTGAGCGACC | F: GCCATGGAGGCCAGTGAATTCATGGGCTGCTTCCACTCAA  R: CAGCTCGAGCTCGATGGATCCTCATGTGGCAATCTCATCG | - |
| BrrCBL1.2 | F: ATGGGCTGCTTCCAATCAAA  R: TCATGTGACAATCTCATCCA | F: ACTTGCCTTGTTCAAGAACCGT  R: CCCGTACAGTCCATGTCGTAAA | F: GCCATGGAGGCCAGTGAATTCATGGGCTGCTTCCAATCAAA  R: CAGCTCGAGCTCGATGGATCCTCATGTGACAATCTCATCCA | - |
| BrrCBL2.1 | F: ATGGCGCAGTGCATAGACGGT  R: TCAGGTATCTTCAACCTGAGA | F: GCTTGTTTGCAGATCGGGTT  R: TAGGCGCATTAGGGTGGAAG | F: GCCATGGAGGCCAGTGAATTCATGGCGCAGTGCATAGACGGT  R: CAGCTCGAGCTCGATGGATCCTCAGGTATCTTCAACCTGAGA | - |
| BrrCBL2.2 | F: ATGGAGAGTTATGATTTGCA  R: TCACTTGAGATATTGAAGAGT | F: ACACAGTGCATAGACGGTGTTA  R: ATTCAGGATCTCCAAGGCCAC | F: GCCATGGAGGCCAGTGAATTCATGGAGAGTTATGATTTGCA  R: CAGCTCGAGCTCGATGGATCCTCACTTGAGATATTGAAGAGT | - |
| BrrCBL3.1 | F: ATGTCGCAGTGCGTAGACG  R: TCAGGTATCTTCCACTTGC | F: TATGCAACTCTTTGTTGCGCT  R: AGCCCATCATCAATCACAGC | F: GCCATGGAGGCCAGTGAATTCATGTCGCAGTGCGTAGACG  R: CAGCTCGAGCTCGATGGATCCTCAGGTATCTTCCACTTGC | - |
| BrrCBL3.2 | F: ATGAGCTCTTTACCGATCTTA  R: TCACTTGAGATAATCCAGAGT | F: CCTCTGCAACAAACCAACCTG  R: GGTGGTGCTTCTTTCGCTTC | F: GCCATGGAGGCCAGTGAATTCATGAGCTCTTTACCGATCTTA  R: CAGCTCGAGCTCGATGGATCCTCACTTGAGATAATCCAGAGT | F: TGGCGCGCCACTAGTGGATCCATGAGCTCTTTACCGATCTTA  R: CATCCCGGGAGCGGTACCTCACTTGAGATAATCCAGAGT |
| BrrCBL3.3 | F: ATGACGCAGTGCGTAGACGG  R: TCATAATACTTTCAAGAGCTG | F: AGTCTGGAGGGCTTGGAGAT  R: GCCCATCATCAATCACAGCAC | F: GCCATGGAGGCCAGTGAATTCATGACGCAGTGCGTAGACGG  R: CAGCTCGAGCTCGATGGATCCTCATAATACTTTCAAGAGCTG | - |
| BrrCBL4.1 | F: ATGGGCTGCGCTCCGTCGAAG  R: GAAATATAGGTTTTGCAACTC | F: CGGTACAGGAAGTGGAAGCG  R: GCGAAAAGGTTCTTCCGGTT | F: GCCATGGAGGCCAGTGAATTCATGGGCTGCGCTCCGTCGAAG  R: CAGCTCGAGCTCGATGGATCCGAAATATAGGTTTTGCAACTC | - |
| BrrCBL4.2 | F: ATGGGCTGCTCTCTGTCGAAG  R: TTAGAAATATAGGTTTTGCAA | F: CGGCAGCAGAAGTTGAAGTTT  R: CCGGTTCCTGTTTCCGAGTAA | F: GCCATGGAGGCCAGTGAATTCATGGGCTGCTCTCTGTCGAAG  R: CAGCTCGAGCTCGATGGATCCTTAGAAATATAGGTTTTGCAA | F: TGGCGCGCCACTAGTGGATCCATGGGCTGCTCTCTGTCGAAG  R: CATCCCGGGAGCGGTACCTTAGAAATATAGGTTTTGCAA |
| BrrCBL4.3 | F: ATGGGTTGCTCTCTGTCAAAG  R: TCACTTTAGATAAGGCAAAGT | F: CTCGGAAACAGGAACCGGAA  R: GGACAGGTGCATTTGGATGG | F: GCCATGGAGGCCAGTGAATTCATGGGTTGCTCTCTGTCAAAG  R: CAGCTCGAGCTCGATGGATCCTCACTTTAGATAAGGCAAAGT | - |
| BrrCBL5 | F: ATGGGATGTGTTTGCAGTAAG  R: TTACTTCAAGAAAGGGATGGT | F: ACGGAGCGGATGTTTGGATT  R: CCTGTGATTCTCTTCCGGTTCA | F: GCCATGGAGGCCAGTGAATTCATGGGATGTGTTTGCAGTAAG  R: CAGCTCGAGCTCGATGGATCCTTACTTCAAGAAAGGGATGGT | - |
| BrrCBL6 | F: ATGATGATGCAGTGTTTAGAT  R: TCATCCAGATGATGGGTTGTC | F: ACGTGCCATTTCGGTTTTCC  R: ATTCAGCTAGAGTCGCCACC | F: GCCATGGAGGCCAGTGAATTCATGATGATGCAGTGTTTAGAT  R: CAGCTCGAGCTCGATGGATCCTCATCCAGATGATGGGTTGTC | - |
| BrrCBL8 | F: ATGTTGGCATTCGTGAAACG  R: TTAGTCGTCTACTTCGGAGT | F: CTTCATCCAGCGCCATGAGT  R: GCGACGAGCTCTTTCCATTC | F: GCCATGGAGGCCAGTGAATTCATGTTGGCATTCGTGAAACG  R: CAGCTCGAGCTCGATGGATCCTTAGTCGTCTACTTCGGAGT | - |
| BrrCBL9.1 | F: ATGGGCTGTTTACATTCCA  R: TCAAGTCGCAATCTCATCC | F: GGCTGTTTACATTCCACGGC  R: GCCATCGTCAACAACTGAGC | F: GCCATGGAGGCCAGTGAATTCATGGGCTGTTTACATTCCA  R: CAGCTCGAGCTCGATGGATCCTCAAGTCGCAATCTCATCC | F: TGGCGCGCCACTAGTGGATCCATGGGCTGTTTACATTCCA  R: CATCCCGGGAGCGGTACCTCAAGTCGCAATCTCATCC |
| BrrCBL9.2 | F: ATGGGCTGTTTACATTCCA  R: TCACGTCGCAATCTCATCA | F: ATTGAGCGCCAAGAGGTGAA  R: TCTTCCCATCCCGATCCACA | F: GCCATGGAGGCCAGTGAATTCATGGGCTGTTTACATTCCA  R: CAGCTCGAGCTCGATGGATCCTCACGTCGCAATCTCATCA | F: TGGCGCGCCACTAGTGGATCCATGGGCTGTTTACATTCCA  R: CATCCCGGGAGCGGTACCTCACGTCGCAATCTCATCA |
| BrrCBL10.1 | F: ATGTGTTCACCTCCACCT  R: TCAGTCCTCAACCTCAGTGTT | F: TGGACTGGACGAACGTTTCC  R: CAAGACGAGTCAGATCCGGG | F: GCCATGGAGGCCAGTGAATTCATGTGTTCACCTCCACCT  R: CAGCTCGAGCTCGATGGATCCTCAGTCCTCAACCTCAGTGTT | - |
| BrrCBL10.2 | F: ATGGACTGGACGAACGTTTCC  R: GGAAATGCCGTCGTCACATCC | F: GAGAGAAAGTCTGCGCCGT  R: CAAGACGAGCCAGATCCACG | F: GCCATGGAGGCCAGTGAATTCATGGACTGGACGAACGTTTCC  R: CAGCTCGAGCTCGATGGATCCGGAAATGCCGTCGTCACATCC | - |
| BrrCBL10.3 | F: ATGCTTCGATTGCCGCCGCCG  R: TCAGTCTTCAACCTCAGTGTT | F: TGTCGGAGAGAAAGTCTGCG  R: CCAGATCCACGTGGTGACAT | F: GCCATGGAGGCCAGTGAATTCATGCTTCGATTGCCGCCGCCG  R: CAGCTCGAGCTCGATGGATCCTCAGTCTTCAACCTCAGTGTT | - |
| BrrCBL10.4 | F: ATGGACTTGCCGGGAGTTTC  R: TTATTTTACCGTCTTTGTCAG | F: TCCACCTCCCATCATGGAGAAT  R: TGCGGCAATCGAAGCATTGT | F: GCCATGGAGGCCAGTGAATTCATGGACTTGCCGGGAGTTTC  R: CAGCTCGAGCTCGATGGATCCTTATTTTACCGTCTTTGTCAG | - |
| BrrCIPK1.1 | F: ATGGTGAGAGAGCAAGCGGA  R: CTATGTTACTAGCTCTTGTT | F: AGATCACTGACTTTGGCCTCA  R: CTCGTAGCCTCTGTTTGCTAGT | F: ATGGCCATGGAGGCCGAATTCATGGTGAGAGAGCAAGCGGA  R: CCGCTGCAGGTCGACGGATCCCTATGTTACTAGCTCTTGTT | - |
| BrrCIPK1.2 | F: ATGGTGAGAAAGCATGAAGAG  R: CTACGTTACTAGTCCTTGTTC | F: GAGAAAGCATGAAGAGGAGGTGA  R: CCGGAGACGGTGTCTTTAGC | F: ATGGCCATGGAGGCCGAATTCATGGTGAGAAAGCATGAAGAG  R: CCGCTGCAGGTCGACGGATCCCTACGTTACTAGTCCTTGTTC | - |
| BrrCIPK2.1 | F: ATGGAGAACAAACCGAGTG  R: CTACAATGGTTCACTATGCT | F: GCACCTTCGCCAAAGTCAAG  R: GTACGGCTCGCTAGAACCTC | F: ATGGCCATGGAGGCCGAATTCATGGAGAACAAACCGAGTG  R: CCGCTGCAGGTCGACGGATCCCTACAATGGTTCACTATGCT | - |
| BrrCIPK2.2 | F: ATGGAGAACAAACCAAGTATA  R: TTAGCCATGCAACACCAGCTC | F: GAGGTTGTTGATTGATGCGGA  R: CCATGCAACACCAGCTCATCC | F: ATGGCCATGGAGGCCGAATTCATGGAGAACAAACCAAGTATA  R: CCGCTGCAGGTCGACGGATCCTTAGCCATGCAACACCAGCTC | - |
| BrrCIPK2.3 | F: ATGGAGAACAAACCAAGCATA  R: CTACAATGGTTCTTGTCCCTC | F: GTGACGGAGACGTTTCACTTG  R: AAACCACGCAACACATGCT | F: ATGGCCATGGAGGCCGAATTCATGGAGAACAAACCAAGCATA  R: CCGCTGCAGGTCGACGGATCCCTACAATGGTTCTTGTCCCTC | - |
| BrrCIPK3 | F: ATGAATCGGAGGCAGCAAG  R: TCACTTTCCAGCTTTCTTCA | F: AATCGGAGGCAGCAAGTCAA  R: TGAGGGCTACAGGTTCTCCA | F: ATGGCCATGGAGGCCGAATTCATGAATCGGAGGCAGCAAG  R: CCGCTGCAGGTCGACGGATCCTCACTTTCCAGCTTTCTTCA | - |
| BrrCIPK4.1 | F: ATGGGCTTTCCATCTCCACC  R: TTAAGCTAAGTCACTTTCAAC | F: CGAATCCGGAAACGAGGATGA  R: CCGATAGATCCAATCCCGCA | F: ATGGCCATGGAGGCCGAATTCATGGGCTTTCCATCTCCACC  R: CCGCTGCAGGTCGACGGATCCTTAAGCTAAGTCACTTTCAAC | - |
| BrrCIPK4.2 | F: ATGTTGCCTCCGTCAACATC  R: TCAACATTTTTTAGATACGTC | F: GCAAATACGAACTCGGTCGC  R: ATCATCGGTTCCATACCGGC | F: ATGGCCATGGAGGCCGAATTCATGTTGCCTCCGTCAACATC  R: CCGCTGCAGGTCGACGGATCCTCAACATTTTTTAGATACGTC | - |
| BrrCIPK5 | F: ATGGAGGAAGAACGTCGAG  R: TTAAACCGTGCCATGACCA | F: GAGCTTGTTCCAGAGCGAGA  R: TTCCTCCCTTCCGTTTTCCC | F: ATGGCCATGGAGGCCGAATTCATGGAGGAAGAACGTCGAG  R: CCGCTGCAGGTCGACGGATCCTTAAACCGTGCCATGACCA | - |
| BrrCIPK6.1 | F: ATGGTCGGAGCAAAACCTATAG  R: TCAAGCTGGTGTAGTAGAAGTC | F: CTGGATGGCTCTCCTCCGAT  R: CTCCTCAGCCTCTGGTGTTG | F: ATGGCCATGGAGGCCGAATTCATGGTCGGAGCAAAACCTATAG  R: CCGCTGCAGGTCGACGGATCCTCAAGCTGGTGTAGTAGAAGTC | - |
| BrrCIPK6.2 | F: ATGGTCGGAGCAAAACCTA  R: AGCAGGCGTAGTAGAAGTC | F: TACGAGCTAGGCCGTCTTCT  R: TGCTTCACCATCCTCATCACC | F: ATGGCCATGGAGGCCGAATTCATGGTCGGAGCAAAACCTA  R: CCGCTGCAGGTCGACGGATCCAGCAGGCGTAGTAGAAGTC | - |
| BrrCIPK6.3 | F: ATGGTCGGAGCTAAACCGGTG  R: AACAGGTGTTGAAGTCCAG | F: AGAAAGGATCTGACGGTGCG  R: GCCATGCTTTTTCCCGTTGT | F: ATGGCCATGGAGGCCGAATTCATGGTCGGAGCTAAACCGGTG  R: CCGCTGCAGGTCGACGGATCCAACAGGTGTTGAAGTCCAG | - |
| BrrCIPK6.4 | F: ATGGTCGGAGCTAAACCAG  R: AGCAGGTGTAGAAGTCCAGAA | F: GTCGTGCGGTGTGATCCTTTTTG  R: GAGCCGTCTCGCATCAGAAGA | F: ATGGCCATGGAGGCCGAATTCATGGTCGGAGCTAAACCAG  R: CCGCTGCAGGTCGACGGATCCAGCAGGTGTAGAAGTCCAGAA | - |
| BrrCIPK7.1 | F: ATGGACTCTCTTCCTCAGCCG  R: TTACATGACGTCATTGTGCCA | F: ATCGTCATGGAACTCGCCTC  R: ACCTTGAGGTTCCCTTCCCT | F: ATGGCCATGGAGGCCGAATTCATGGACTCTCTTCCTCAGCCG  R: CCGCTGCAGGTCGACGGATCCTTACATGACGTCATTGTGCCA | F: TGGCGCGCCACTAGTGGATCCATGGACTCTCTTCCTCAGCCG  R: CATCCCGGGAGCGGTACCTTACATGACGTCATTGTGCCA |
| BrrCIPK7.2 | F: ATGGACTCCCTTCCTCAACCG  R: TTACATGACGTCATTGTGCCA | F: GCGGTCGTCTTGCCGAA  R: AAGCGGATAAGCCGAAGTCC | F: ATGGCCATGGAGGCCGAATTCATGGACTCCCTTCCTCAACCG  R: CCGCTGCAGGTCGACGGATCCTTACATGACGTCATTGTGCCA | - |
| BrrCIPK8 | F: ATGGTGGTGGTAAGAAAAGTA  R: TCAACGTCTCTTACTCTTGGT | F: GCACCTTCGCCAAAGTCAAG  R: GTACGGCTCGCTAGAACCTC | F: ATGGCCATGGAGGCCGAATTCATGGTGGTGGTAAGAAAAGTA  R: CCGCTGCAGGTCGACGGATCCTCAACGTCTCTTACTCTTGGT | - |
| BrrCIPK9.1 | F: ATGAGCGGGAGTAGAAAGAAG  R: TTAAGCTTTATGTTCTTCAGG | F: ACCGGAATCCATGAACGCTT  R:CTCGCAGGTCGTTGAGAAGTA | F: ATGAGCGGGAGTAGAAAGAAG  R: CCGCTGCAGGTCGACGGATCCTTAAGCTTTATGTTCTTCAGG | - |
| BrrCIPK9.2 | F: ATGGGACGAACTCTAGGCG  R: R TTAAACTTTATGTTCTTGA | F: GCAGCAGCAGATGTATGGTCA  R: TGACATTCTTGGCACCTGGC | F: ATGGCCATGGAGGCCGAATTCATGGGACGAACTCTAGGCG  R: CCGCTGCAGGTCGACGGATCCRTTAAACTTTATGTTCTTGA | - |
| BrrCIPK10.1 | F: ATGGAAAATAAGCCGAGTG  R: CTACAATGGTTCTTCTTCT | F: TTAAGCGCCCTTGCCGATT  R: TGCCTTTGTCCCATCGTAGC | F: ATGGCCATGGAGGCCGAATTCATGGAAAATAAGCCGAGTG  R: CCGCTGCAGGTCGACGGATCCCTACAATGGTTCTTCTTCT | - |
| BrrCIPK10.2 | F: ATGGAAAACAAACCAAGTG  R: CAACGATTCTTCTTCCTGTTT | F: ACGCCGACGTTTCATATGGT  R: TGCTGTGAAGCAGACACCAA | F: ATGGCCATGGAGGCCGAATTCATGGAAAACAAACCAAGTG  R: CCGCTGCAGGTCGACGGATCCCAACGATTCTTCTTCCTGTTT | - |
| BrrCIPK11.1 | F: ATGCCGGAGATCGAGATTGTC  R: AATACTTGCGTTTGGCGTTTG | F: AGGGCAAGGAAGATGGAGGA  R: GCGACTTCTCCGAGAGGAAC | F: ATGGCCATGGAGGCCGAATTCATGCCGGAGATCGAGATTGTC  R: CCGCTGCAGGTCGACGGATCCAATACTTGCGTTTGGCGTTTG | - |
| BrrCIPK11.2 | F: ATGTCGGAGATCGAGGTTGTC  R: TGATAAATCCACAGCTGCG | F: TCGGGGTGTATATTTCGCGG  R: GATCGCAAACGCGTTCAAGT | F: ATGGCCATGGAGGCCGAATTCATGTCGGAGATCGAGGTTGTC  R: CCGCTGCAGGTCGACGGATCCTGATAAATCCACAGCTGCG | - |
| BrrCIPK11.3 | F: ATGCCGGAGATCCAGATTGTC  R: AATAGTTGCGATTGGCGTTTG | F: ACAACAGCAACACCAATGCC  R: TGAGGATCTTGATGGCGACG | F: ATGGCCATGGAGGCCGAATTCATGCCGGAGATCCAGATTGTC  R: CCGCTGCAGGTCGACGGATCCAATAGTTGCGATTGGCGTTTG | - |
| BrrCIPK11.4 | F: ATGCGCCGTTTATCTCACCC  R: AATACTTGCGTTTGGCGTTTG | F: CGGTGCCCTAGATGGATGTC  R: ATCTTCCTTGCCCTTCACGG | F: ATGGCCATGGAGGCCGAATTCATGCGCCGTTTATCTCACCC  R: CCGCTGCAGGTCGACGGATCCAATACTTGCGTTTGGCGTTTG | - |
| BrrCIPK12.1 | F: ATGGCGGAGCTAACGAAAG  R: CTACTCAGTGTCAGAAGGC | F: GGCGGAGCTAACGAAAGAAAC  R: TCGTTTGTCTTGACGTTGCG | F: ATGGCCATGGAGGCCGAATTCATGGCGGAGCTAACGAAAG  R: CCGCTGCAGGTCGACGGATCCCTACTCAGTGTCAGAAGGC | - |
| BrrCIPK12.2 | F: ATGTCGGATCTCAAAAGAGAA  R: CTATTCAGTATCAGAAGGCAA | F: GTGTTTGGAGCCGAGAAGGA  R: CGAAACCGGAGCTCCTGAAA | F: ATGGCCATGGAGGCCGAATTCATGTCGGATCTCAAAAGAGAA  R: CCGCTGCAGGTCGACGGATCCCTATTCAGTATCAGAAGGCAA | - |
| BrrCIPK13.1 | F: ATGGCTCAAGTTCTATCTC  R: TTAACATAGTCTCTCTTCA | F: CCGGGTTTGATCTTTCCGGTTT  R: CGCCTTCGCAATCTCTTCAAGTT | F: ATGGCCATGGAGGCCGAATTCATGGCTCAAGTTCTATCTC  R: CCGCTGCAGGTCGACGGATCCTTAACATAGTCTCTCTTCA | - |
| BrrCIPK13.2 | F: ATGGCTCCAGTACCATCTCC  R: TTAACATGGGACCAAACACA | F: CCAGTACCATCTCCGCAAGT  R: AGGATAGAGCCTTGTGGGGT | F: ATGGCCATGGAGGCCGAATTCATGGCTCCAGTACCATCTCC  R: CCGCTGCAGGTCGACGGATCCTTAACATGGGACCAAACACA | - |
| BrrCIPK13.3 | F: ATGGCTCTGCGTCTTCTTG  R: TCATCTCTCTTCAGTTTCA | F: TCCGCCACCCTTACATTGTC  R: GACGAAGCCGCCCTTTAGAT | F: ATGGCCATGGAGGCCGAATTCATGGCTCTGCGTCTTCTTG  R: CCGCTGCAGGTCGACGGATCCTCATCTCTCTTCAGTTTCA | - |
| BrrCIPK14 | F: ATGCACCGCTTGCGTCACCCC  R: AGTCGTCGTATTCACGTCAC | F: GTGCTCAACGCAGGGTATCT  R: GATTGGTGTCGAGAAGGCGA | F: ATGGCCATGGAGGCCGAATTCATGCACCGCTTGCGTCACCCC  R: CCGCTGCAGGTCGACGGATCCAGTCGTCGTATTCACGTCAC | - |
| BrrCIPK15 | F: ATGGAGCACGTTAAGGGCGGT  R: TCAGTGCCAAGCCAATACAAT | F: TTGGATCCTAACCCCAACGC  R: CTCTGTGTCGTCGGTCTCTG | F: ATGGCCATGGAGGCCGAATTCATGGAGCACGTTAAGGGCGGT  R: CCGCTGCAGGTCGACGGATCCTCAGTGCCAAGCCAATACAAT | - |
| BrrCIPK16 | F: ATGGAAGAATCCAAAACTG  R: TTAATTTATTAAGTTATCA | F: TGTCGGATCCTGAACAACGG  R:TGGAGATGGGAGTGTCTCGT | F: ATGGCCATGGAGGCCGAATTCATGGAAGAATCCAAAACTG  R: CCGCTGCAGGTCGACGGATCCTTAATTTATTAAGTTATCA | F: TGGCGCGCCACTAGTGGATCCATGGAAGAATCCAAAACTG  R: CATCCCGGGAGCGGTACCTTAATTTATTAAGTTATCA |
| BrrCIPK17.1 | F: ATGGTGACGGAAGGAATGC  R: TCTTTGGGATGATGCGTCAA | F: GAGAACGCTTGGTGAAGGGA  R: AAGGGACATTGAGGCGACTG | F: ATGGCCATGGAGGCCGAATTCATGGTGACGGAAGGAATGC  R: CCGCTGCAGGTCGACGGATCCTCTTTGGGATGATGCGTCAA | - |
| BrrCIPK17.2 | F: ATGGTGACGGAAGGAATGCGT  R: TCATCTTTGGGATGATGCGTC | F: GAGAACGCTTGGTGAAGGGA  R: AAGGGACATTGAGGCGACTG | F: ATGGCCATGGAGGCCGAATTCATGGTGACGGAAGGAATGCGT  R: CCGCTGCAGGTCGACGGATCCTCATCTTTGGGATGATGCGTC | - |
| BrrCIPK17.3 | F: ATGGTGACGAAGGAAATGCG  R: CTACCTTAAAAGCTGTTGTA | F: ATGATACAAACCTGGCGGCT  R: TCATGGCAATTGGACGGAGT | F: ATGGCCATGGAGGCCGAATTCATGGTGACGAAGGAAATGCG  R: CCGCTGCAGGTCGACGGATCCCTACCTTAAAAGCTGTTGTA | - |
| BrrCIPK17.4 | F: ATGTCGTTGATAATCGACTTG  R: TTAAAGCTTTTGAACATGAGA | F: TGTCGTTGATAATCGACTTGCAG  R:TGTTCGTCCTTCTGTTTCCGA | F: ATGGCCATGGAGGCCGAATTCATGTCGTTGATAATCGACTTG  R: CCGCTGCAGGTCGACGGATCCTTAAAGCTTTTGAACATGAGA | - |
| BrrCIPK18 | F: ATGTCTCAGGCCTTGGCTCCA  R: CTATACAGTATCACACGGCAA | F: AACATAAGCCCACAGAGCCC  R: CGACGTTTTCGCCGGATTTT | F: ATGGCCATGGAGGCCGAATTCATGTCTCAGGCCTTGGCTCCA  R: CCGCTGCAGGTCGACGGATCCCTATACAGTATCACACGGCAA | - |
| BrrCIPK19 | F: ATGGCGGAATTGCTGAGAA  R: CTAATCAGTATTAGAAAGC | F: AGGAGACTTCTGGTGCCCTA  R: TCAGCGCTAGAGAGCTTGTG | F: ATGGCCATGGAGGCCGAATTCATGGCGGAATTGCTGAGAA  R: CCGCTGCAGGTCGACGGATCCCTAATCAGTATTAGAAAGC | - |
| BrrCIPK20.1 | F: ATGGATAAAAAAGGCATCG  R: TTATGCAACACTCTCATTG | F: GTCACCCAAACGTCGTGTTC  R: ACGGTGGTAAACTCCACGAC | F: ATGGCCATGGAGGCCGAATTCATGGATAAAAAAGGCATCG  R: CCGCTGCAGGTCGACGGATCCTTATGCAACACTCTCATTG | F: TGGCGCGCCACTAGTGGATCCATGGATAAAAAAGGCATCG  R: CATCCCGGGAGCGGTACCTTATGCAACACTCTCATTG |
| BrrCIPK20.2 | F: ATGGATAAAAACGGTATTGT  R: TTCCTTGCCATTTCCAAACA | F: TGGAACACCTGCTTACGTGG  R: AGGAGGAAACCAGTTAGGGC | F: ATGGCCATGGAGGCCGAATTCATGGATAAAAACGGTATTGT  R: CCGCTGCAGGTCGACGGATCCTTCCTTGCCATTTCCAAACA | - |
| BrrCIPK21.1 | F: ATGGGTTTGTTTGGGACCAAG  R: TTAGCTTACTTCGGCTGTAAG | F: GAACTTCGCAAAGGTGAAGCTA  R: GCACGATGTTGGGATGGTTTA | F: ATGGCCATGGAGGCCGAATTCATGGGTTTGTTTGGGACCAAG  R: CCGCTGCAGGTCGACGGATCCTTAGCTTACTTCGGCTGTAAG | - |
| BrrCIPK21.2 | F: ATGGGTTTGTTTGGGACGAAG  R: TTAGCTTACTTCCGCCGTAAG | F: GAACTTCGCAAAGGTGAAGCTA  R: TTTGTCTTGGTCCCAATCACCT | F: ATGGCCATGGAGGCCGAATTCATGGGTTTGTTTGGGACGAAG  R: CCGCTGCAGGTCGACGGATCCTTAGCTTACTTCCGCCGTAAG | - |
| BrrCIPK22.1 | F: ATGGCCGAAGAAGACTCTTC  R: TTACGGTTTATCAGGAACTC | F: GGATGCTGCACACTCTCTGT  R: GTATTGCGCCATGCGGATTT | F: ATGGCCATGGAGGCCGAATTCATGGCCGAAGAAGACTCTTC  R: CCGCTGCAGGTCGACGGATCCTTACGGTTTATCAGGAACTC | - |
| BrrCIPK22.2 | F: ATGGCCGGCGAAGACTCTTC  R: CTACGGACCCCTATTAACCG | F: CGGAGGATCTCCACAACGAC  R: GCCAGCTCCATGACGAAGTA | F: ATGGCCATGGAGGCCGAATTCATGGCCGGCGAAGACTCTTC  R: CCGCTGCAGGTCGACGGATCCCTACGGACCCCTATTAACCG | - |
| BrrCIPK23.1 | F: ATGGCTTCTCGAACAACACC  R: TCAAGAAGCAGCCACAGTAC | F: CGACACCTTCAGGTAGTAGCAG  R: AGCCACACTCTCTCCCTTGT | F: ATGGCCATGGAGGCCGAATTCATGGCTTCTCGAACAACACC  R: CCGCTGCAGGTCGACGGATCCTCAAGAAGCAGCCACAGTAC | - |
| BrrCIPK23.2 | F: ATGGCTTCTCGAGCAACACC  R: TCTTTGGGATGATGCGTCAA | F: GGGCCAGTTAGCAGTCACAA  R: CACCGCACCATTAGTATCACCT | F: ATGGCCATGGAGGCCGAATTCATGGCTTCTCGAGCAACACC  R: CCGCTGCAGGTCGACGGATCCTCTTTGGGATGATGCGTCAA | F: TGGCGCGCCACTAGTGGATCCATGGCTTCTCGAGCAACACC  R: CATCCCGGGAGCGGTACCTCTTTGGGATGATGCGTCAA |
| BrrCIPK23.3 | F: ATGGCCAGCAAAACTAAGATC  R: TCAAGAAGCAACCACCGCACC | F: ATTAGCAGTCACGGCTGAGG  R: GCACCACCTGAAACTTGGGT | F: ATGGCCATGGAGGCCGAATTCATGGCCAGCAAAACTAAGATC  R: CCGCTGCAGGTCGACGGATCCTCAAGAAGCAACCACCGCACC | F: TGGCGCGCCACTAGTGGATCCATGGCCAGCAAAACTAAGATC  R: CATCCCGGGAGCGGTACCAAACGTGATTGTTCTGAATAG |
| BrrCIPK24 | F: ATGGATCAGAAGAAAAGAAT  R: AAACGTGATTGTTCTGAATAG | F: GTGGTTCTCTGCAGACGTGA  R: CTTCGCTCGAGTAGGCATGT | F: ATGGCCATGGAGGCCGAATTCATGGATCAGAAGAAAAGAAT  R: CCGCTGCAGGTCGACGGATCCAAACGTGATTGTTCTGAATAG | - |
| BrrCIPK25 | F: ATGGAGGAAGAACGGCGCGTT  R: TTATATTCTCTCACAGTCACT | F: CTCCATGGTTTTCCCCCGAG  R: CCGTCGTTAAGGACCGTGAT | F: ATGGCCATGGAGGCCGAATTCATGGAGGAAGAACGGCGCGTT  R: CCGCTGCAGGTCGACGGATCCTTATATTCTCTCACAGTCACT | - |
| BrrCIPK26.1 | F:ATGAATCGGCCCAAGGTAC  R: TTTGTTGTTTAGACCAGAGC | F: CTTCACACTGCATGCGGAAC  R: ATTCAGCAGCTGTTATCTGGT | F: ATGGCCATGGAGGCCGAATTCATGAATCGGCCCAAGGTAC  R: CCGCTGCAGGTCGACGGATCCTTTGTTGTTTAGACCAGAGC | - |
| BrrCIPK26.2 | F: ATGAATCGGCCAAAGGTCC  R: TTTGTTGTTATTATCAGAGG | F: GCAGACTTGTGGTCCTGTGG  R: ACGAGTTGTCGGGTTTGGG | F: ATGGCCATGGAGGCCGAATTCATGAATCGGCCAAAGGTCC  R: CCGCTGCAGGTCGACGGATCCTTTGTTGTTATTATCAGAGG | - |

**Cloning, primers for BrrCBLs and BrrCIPKs amplified; Expression, primers for qRT-PCR analysis; Y2H, primers for yeast two-hybrid assay; BiFC, primers for bimolecular fluorescence complementation assays**
