# Supplementary figures and images for: Genome-Wide Identification and Functional Analysis of the Calcineurin B-like Protein and Calcineurin B-like Protein-Interacting Protein Kinase Gene Families in Turnip (Brassica rapa var. rapa)
Source: Front Plant Sci. 2017 Jul 7;8:1191. doi: 10.3389/fpls.2017.01191 (PMC5500646; doi:10.3389/fpls.2017.01191)

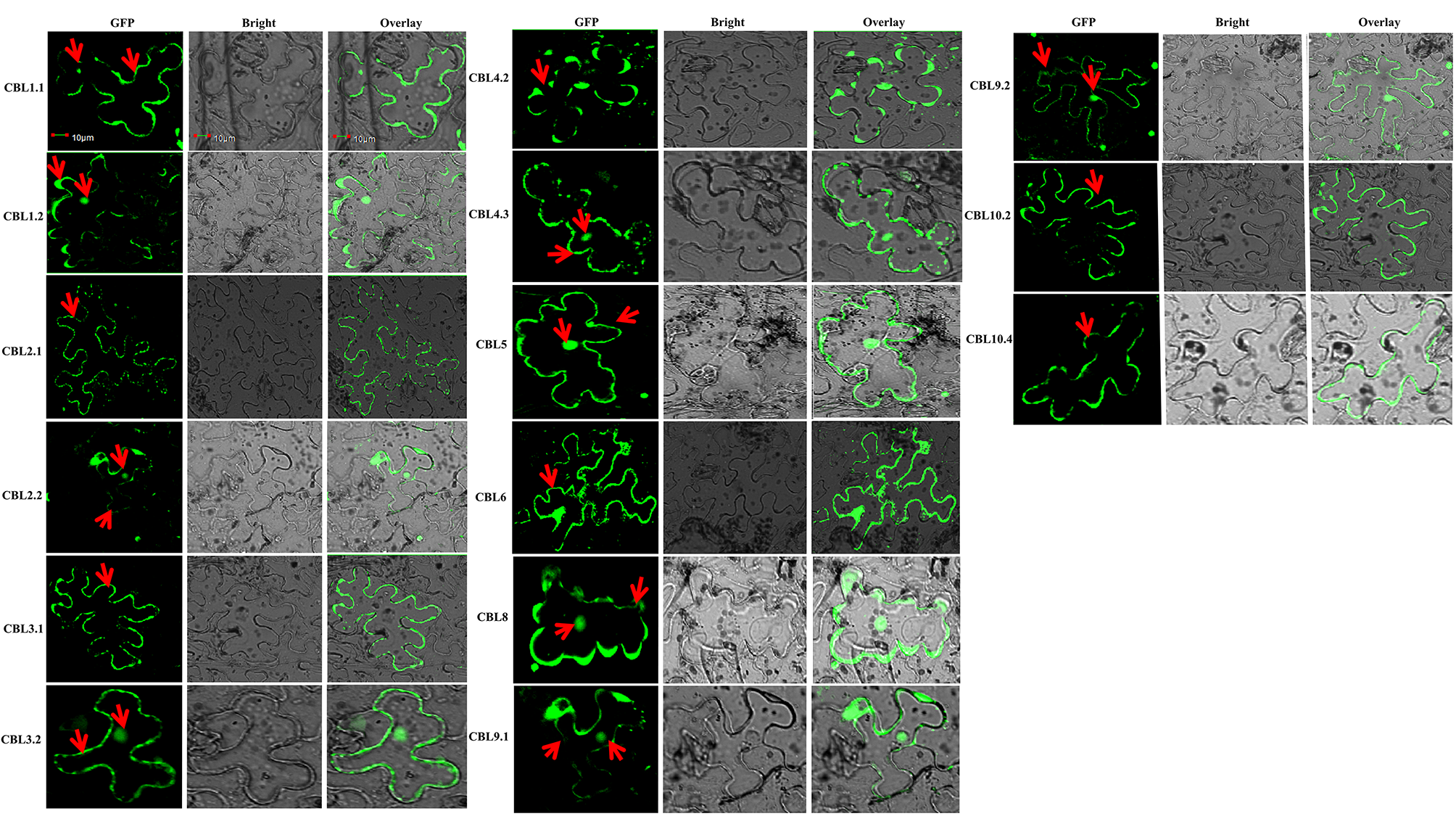

Supplement: Supplementary Figure 1 — Subcellular localization of 15 35S: BrrCBL-GFP fusion protein in N. benthamiana leaf cells. Bar = 10 μm. [file Image1.TIF]

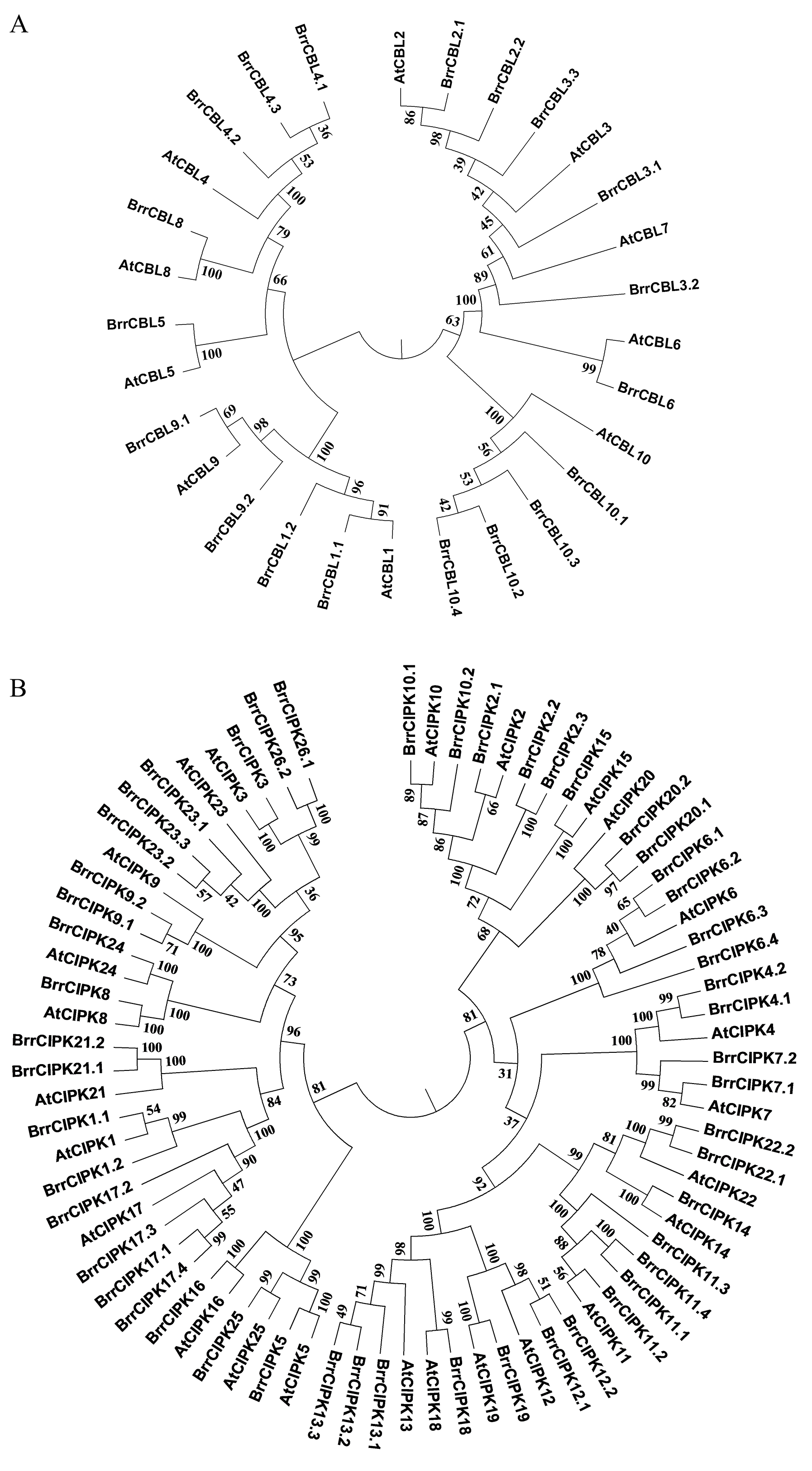

Supplement: Supplementary Figure 2 — Phylogenetic relationships of BrrCBL and BrrCIPK proteins in Arabidopsis and Brassica rapa var. rapa. (A) Phylogenetic relationships of BrrCBL proteins in Arabidopsis and Brassica rapa var. rapa. (B) Phylogenetic relationships of BrrCIPK proteins in Arabidopsis and Brassica rapa var. rapa. The protein sequences were aligned using the MAFFT version 7 program, and phylogenetic trees were constructed using the MEGA 5.0 software with the neighbour-joining method and the 1,000 bootstrap test replicates. [file Image2.TIF]

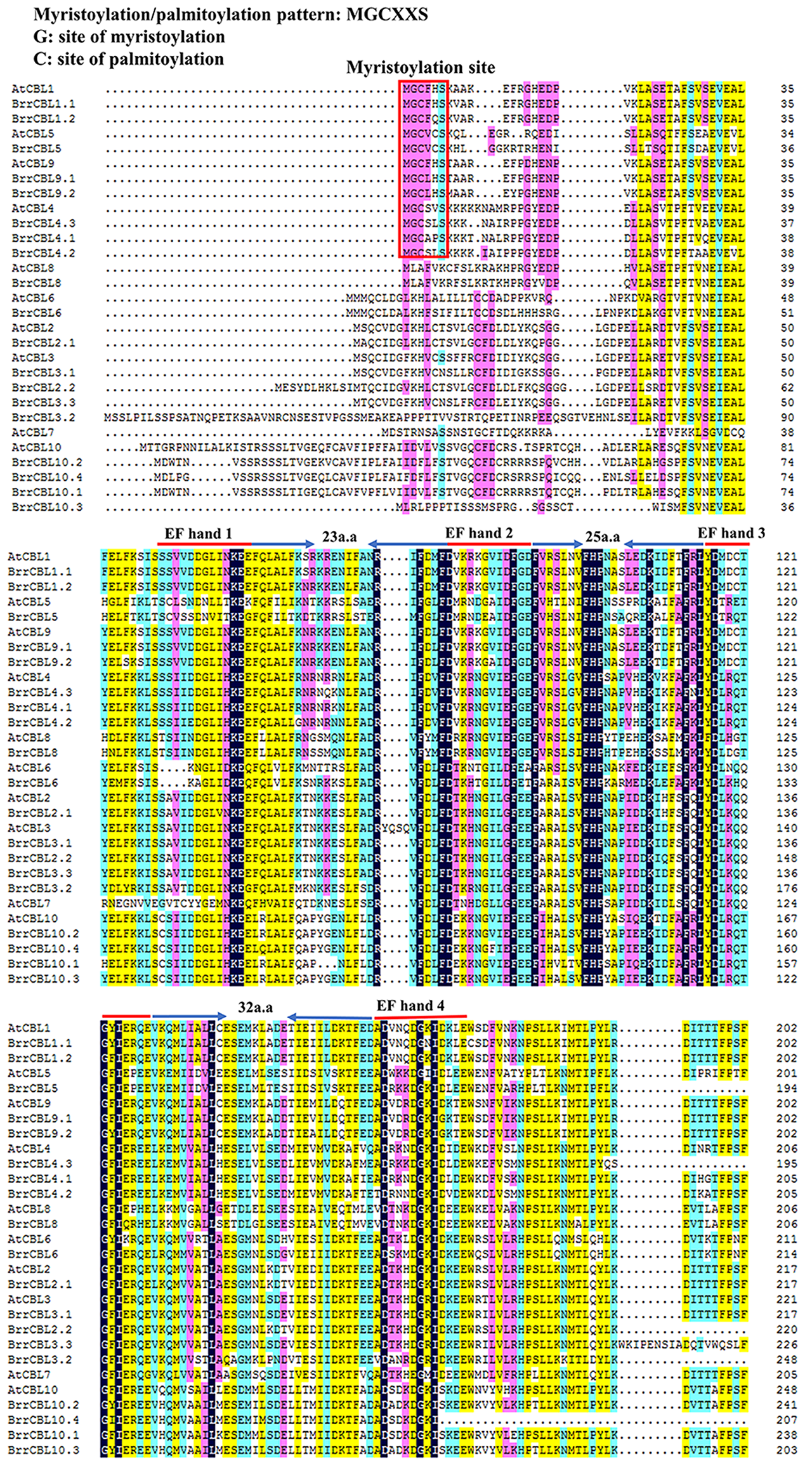

Supplement: Supplementary Figure 3 — Multiple sequence alignments of the amino acids of 19 BrrCBLs and 10 Arabidopsis CBLs. The myristoylation and palmitoylation site was the red rectangle. The four EF-hand conserved domains were all indicated with straight lines. [file Image3.TIF]

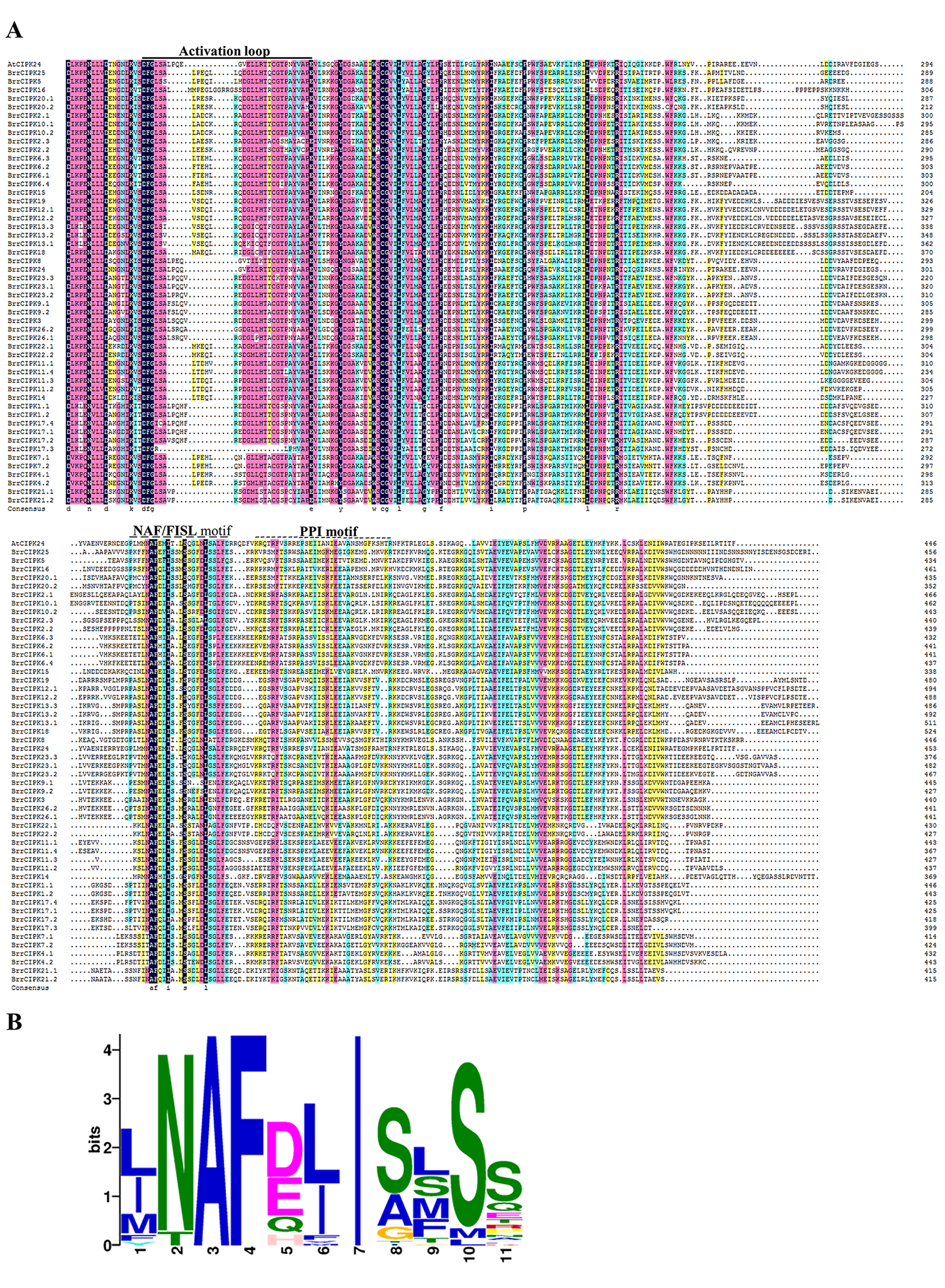

Supplement: Supplementary Figure 4 — Multiple sequence alignments of the amino acids of 51 turnip BrrCIPKs and Arabidopsis CIPK24. (A) CIPK proteins consist of a conserved N-terminal kinase domain, and a C-terminal regulatory domain. The activation loop was denoted by straight lines. The conserved NAF/FISL motif within the rather divergent C-terminal regulatory domain was denoted by dashed lines. The protein-phosphatase interaction (PPI) motif within the C-terminus of these kinases was marked by dots above the sequences. (B) An MEME analysis of the NAF/FISL motif in 51 BrrCIPKs. [file Image4.TIF]

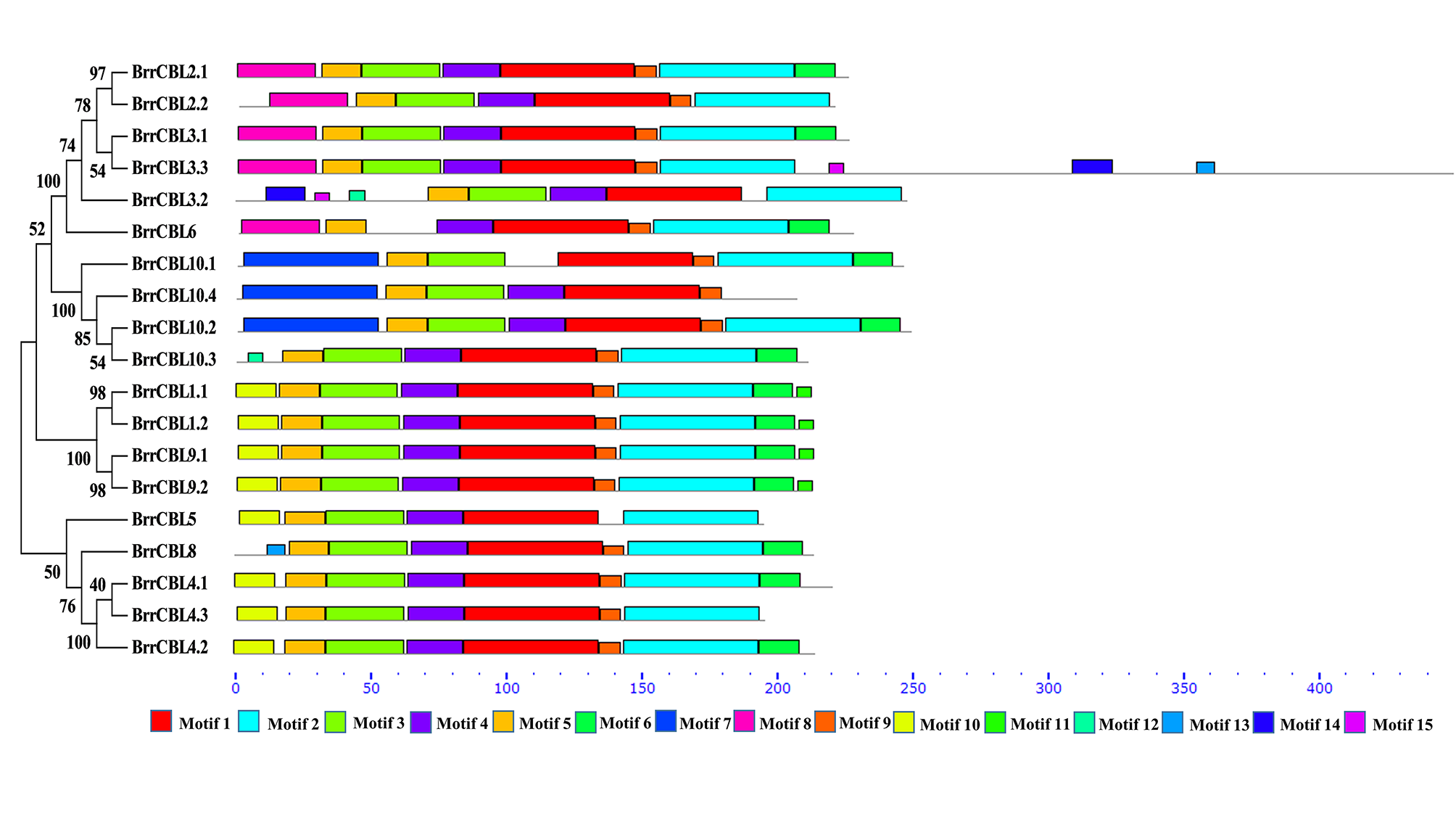

Supplement: Supplementary Figure 5 — An MEME analysis of motif compositions of BrrCBLs. A total of 15 conserved motifs designated as motif 1 to motif 15 were identified within the genes. [file Image5.TIF]

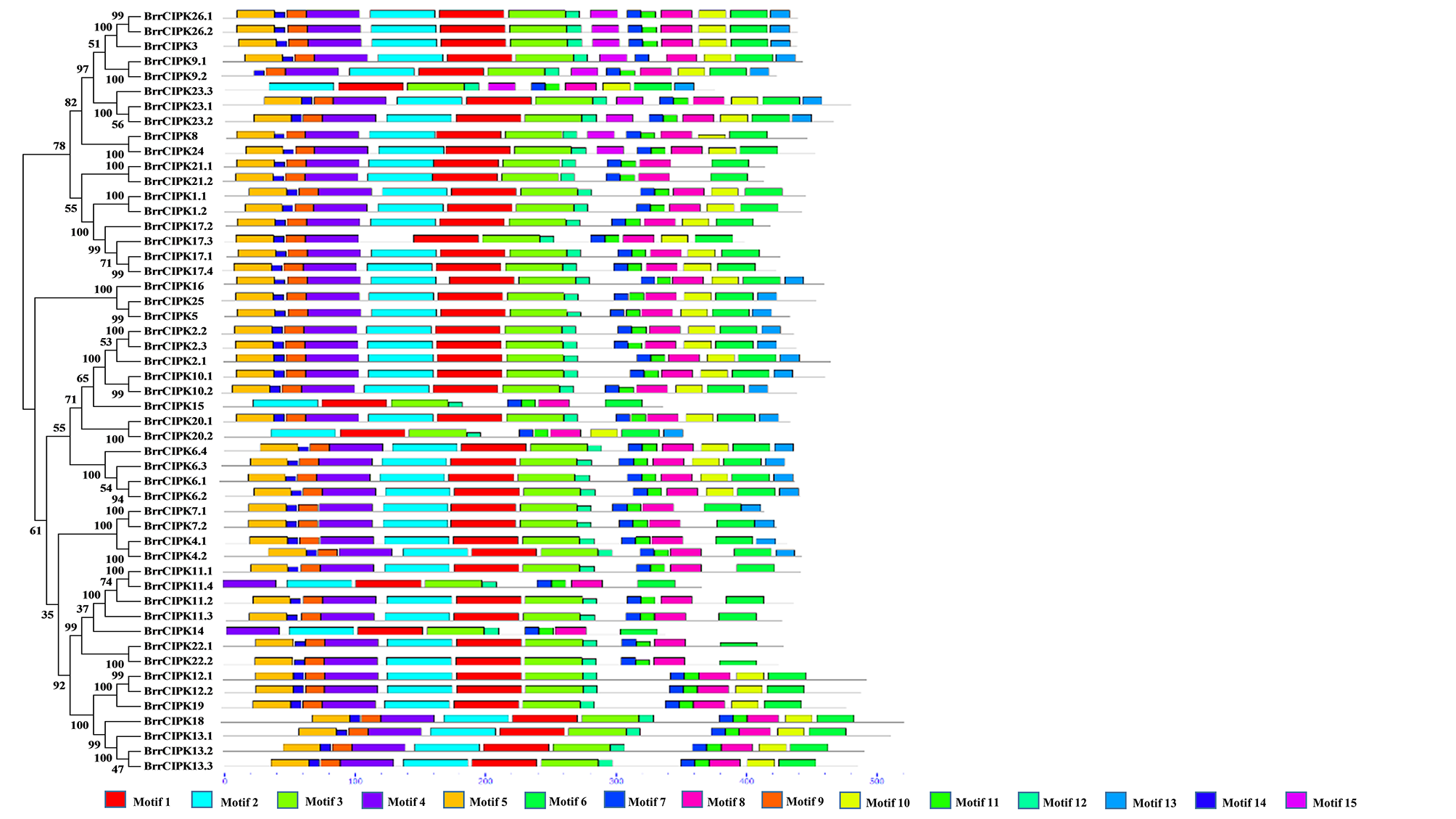

Supplement: Supplementary Figure 6 — An MEME analysis of motif compositions of BrrCIPKs. A total of 15 conserved motifs designated as motif 1 to motif 15 were identified within the genes. [file Image6.TIF]

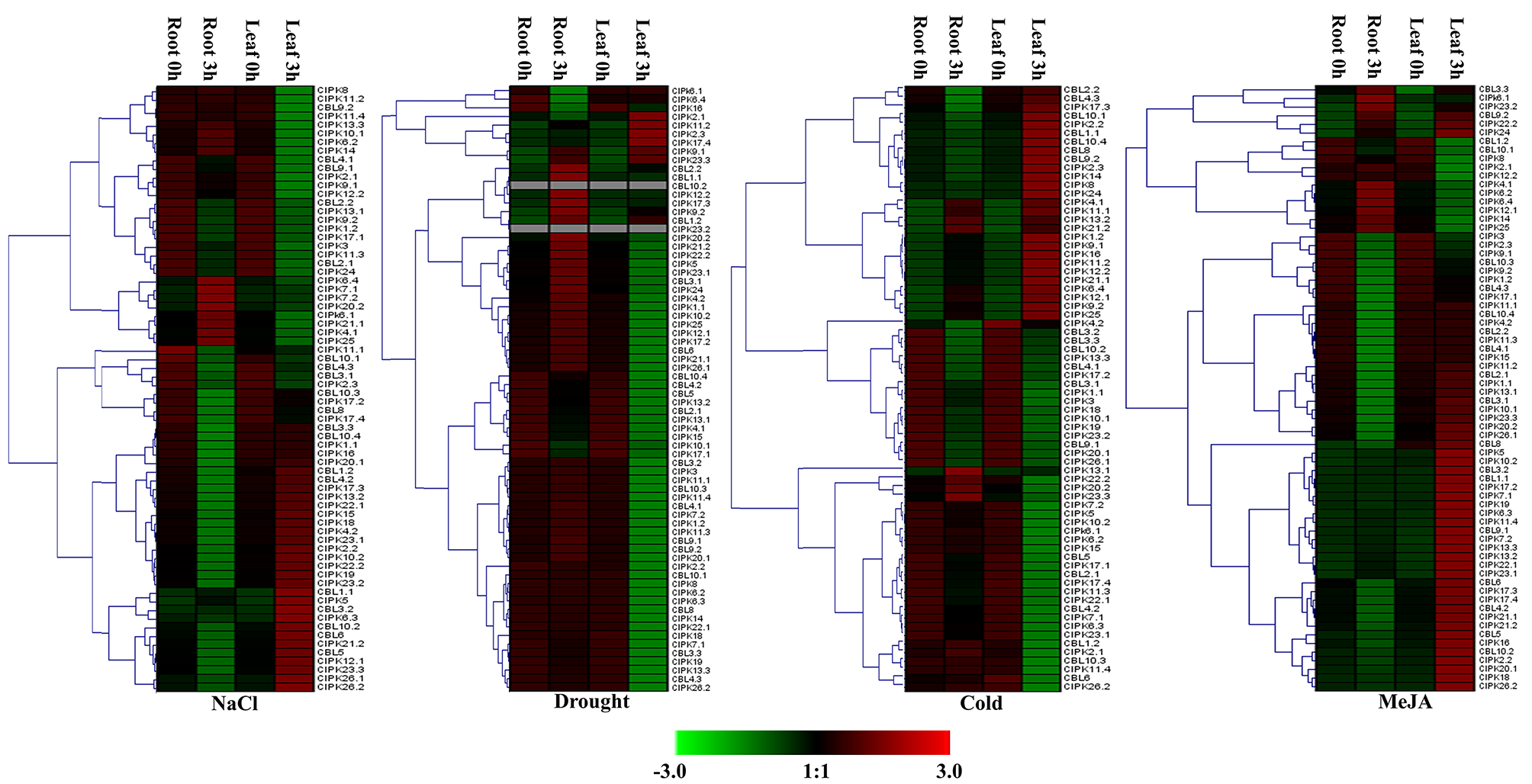

Supplement: Supplementary Figure 7 — The co-expression analysis among BrrCBLs and BrrCIPKs under NaCl, drought, cold, and MeJA treatment, respectively. [file Image7.TIF]
